# USP11 deubiquitinates GATA3 to suppress the growth and metastasis of luminal-type breast cancers

**DOI:** 10.1016/j.jbc.2026.113371

**Published:** 2026-07-29

**Authors:** Tao Qian, Xiong Liu, Feng Bai, Qiyue He, Yipei Xu, Jiayi Zhang, Shiwen Zhang, Shaoling Ye, Yuchan Wang, Bin Peng, Kapanova Gulnara, Jasur Rizaev, Wei-Guo Zhu, Xingzhi Xu, Xin-Hai Pei

**Affiliations:** 1 Guangdong Provincial Key Laboratory of Infection Immunity and Inflammation, International Cancer Center, Marshall Laboratory of Biomedical Engineering, Shenzhen University Medical School, Shenzhen University General Hospital, Shenzhen, Guangdong, China; 2 Department of Pathology, Shenzhen University Medical School, Shenzhen, Guangdong, China; 3 Gansu Dian Medical Laboratory, Lanzhou, China; 4 Guangdong Key Laboratory for Genome Stability & Disease Prevention and International Cancer Center, Shenzhen University Medical School, Shenzhen, Guangdong, China; 5 Shenzhen-Almaty Joint Laboratory for Frontiers in Medical Science (salFMS), Shenzhen University Medical School, Shenzhen, Guangdong, China; 6 Department of Health Policy and Organization, Al-Farabi Kazakh National University, Almaty, Kazakhstan; 7 Department of Public Health and Healthcare Management, Samarkand State Medical University, Samarkand, Uzbekistan; 8 Department of Biochemistry and Molecular Biology, International Cancer Center, Shenzhen University Medical School, Shenzhen, Guangdong, China; 9 Department of Anatomy and Histology, Shenzhen University Medical School, Shenzhen, Guangdong, China

**Keywords:** ERα, GATA3, luminal-type breast cancer, p27, USP11

## Abstract

Luminal breast cancers account for 70%-80% of total breast cancer cases. More than half of luminal breast cancers either relapse after treatment or lose their luminal features, resulting in poorly differentiated cancers with enhanced invasiveness and metastasis, yet the mechanisms remain largely elusive. In this study, we demonstrated that Ubiquitin-specific protease 11 (USP11) suppresses the proliferation and migration of luminal epithelial and tumor cells *in vitro*, but not basal-like tumor cells. Consistently, USP11 inhibits luminal tumorigenesis and metastasis, but promotes basal-like tumorigenesis *in vivo*. Mechanistically, USP11 positively regulates GATA3, p27, and ERα in a deubiquitinase activity-dependent manner. USP11 deubiquitinates GATA3, which subsequently binds to and transcriptionally activates *CDKN1B* (encoding p27). USP11 stimulates *CDKN1B* and *ESR1* (encoding ERα) transcription in a GATA3-dependent manner. GATA3 knockdown reverses the increase in p27 and ERα and the decrease in cell proliferation and migration induced by USP11 overexpression, and p27 knockdown rescues the proliferative inhibition induced by USP11 or GATA3 overexpression in luminal tumorigenesis. USP11 overexpression sensitizes luminal breast cancer cells to tamoxifen treatment. The expression levels of USP11, GATA3, p27, and ERα are positively correlated with each other in human breast tumors and cell lines. We thus identify a novel USP11-GATA3 axis that governs luminal cell fate and restrains luminal cell proliferation and migration during mammary tumor development and metastasis.

Breast cancers are classified by gene expression profiling into five intrinsic subtypes: luminal A, luminal B, HER2-enriched, basal-like, and normal-like. Luminal tumors are characterized by estrogen receptor (ER) expression, whereas basal-like tumors are typically ER-negative. Basal-like breast cancers are highly invasive, poorly differentiated, and contain several distinct cell types such as cells that express luminal, basal, and mesenchymal biomarkers (1, 2). Some basal-like breast cancers are thought to originate from luminal epithelial cells or arise through transdifferentiation of luminal cancer cells (3, 4, 5, 6, 7). Luminal breast cancers, accounting for approximately 70%∼80% of total breast cancer cases diagnosed, are relatively well-differentiated and, in most cases, considered as the initiation and early stage of tumor progression (8). Relative to basal-like breast cancers, most luminal breast cancers proliferate slowly, but respond to targeted hormone therapy. Because of the targeted therapy-induced drug resistance and yet unknown mechanisms, more than half of luminal breast cancers either relapse after treatment or lose their luminal features, resulting in poorly differentiated cancers with enhanced invasiveness and metastasis (9). It remains largely elusive how luminal cell fate is maintained and how luminal cell proliferation is controlled in the mammary gland and breast cancer.

Ubiquitin-specific protease 11 (USP11) belongs to the deubiquitinating enzyme family, which comprises more than 100 members that remove ubiquitin from target proteins and regulate protein stability, localization, and function (10). USP11 functions in various processes, such as proliferation, differentiation, and metastasis, and is implicated in multiple diseases, including cancer (11). The role of USP11 in regulating tumorigenesis, as well as luminal or epithelial cell differentiation and migration, is contradictory. USP11 may promote epithelial-mesenchymal transition (EMT) *in vitro* by deubiquitination of TGFBR2 and SNAIL; conversely, it may preserve luminal or epithelial cell characteristics through the stabilization of phosphatase and tensin homolog (PTEN) and positive regulation of ERα transcriptional activity (11, 12). USP11 deubiquitinates PPP1CA, E2F1, NF90, Snail, NONO, Nrf2, SREBF1, AR, and MYC to promote tumorigenesis; however, it also removes the ubiquitin chain from PTEN, ARID1A, RhoA, PML, or the cell cycle inhibitor p21^Cdkn1a^ (p21) to inhibit tumorigenesis (13, 14, 15, 16, 17, 18, 19, 20, 21, 22, 23, 24, 25). The pro- or anti-tumor effect of USP11 depends primarily on the function of its substrates in different cancer types or even within the same type of cancer. The role of USP11 in breast cancer is also complex. It facilitates breast cancer progression through deubiquitinating XIAP, TGF-βR2, cytoplasmic p21, or PGAM5; yet it may postpone this process by deubiquitinating PTEN ((11, 12, 22, 26, 27, 28)). We recently discovered that USP11 deubiquitinates E-cadherin (E-cad), P-cadherin, and N-cadherin, which play a critical role in breast cancer (29). However, most, if not all, findings regarding USP11 in mammary cell proliferation have been derived from cell culture-based systems. Currently, no genetic or molecular evidence indicates that USP11 controls the proliferation and migration of luminal cells in the mammary gland or in tumors.

Clinically, the expression of the transcription factor GATA3 is a key feature of luminal breast cancers (30). Gata3 is required for mammary luminal epithelial differentiation and mammary gland development (31, 32). Germline or epithelium-specific deletion of *Gata3* in mice causes early lethality or severe growth defects (31, 32, 33, 34). Overexpression of GATA3 suppresses epithelial-mesenchymal transition in cancer cell lines (35, 36, 37). Loss of Gata3 reduces the expression of ERα and induces basal-like mammary tumors (7, 31); it also marks loss of differentiation, progression of luminal mammary tumors, and gain of metastatic potential (38, 39). However, targeted deletion of GATA3 in early luminal tumors led to cell apoptosis (38). The function of GATA3 in controlling cell proliferation is puzzling and likely cell-type dependent. Deficiency of Gata3 impairs T cell proliferation (40, 41, 42), but stimulates B cell proliferation and promotes B cell lymphomagenesis (42). Deficiency of Gata3 reduces the proliferation of mammary luminal epithelial cells (7, 43) with induction of cell cycle inhibitor *p18*^*Ink4c*^ (*p18*); however, deficiency of Gata3 in a p18^Ink4c^-null background promotes mammary tumorigenesis. Overexpression of GATA3 in basal-like breast cancer cells (BC) suppresses proliferation and metastasis (44, 45). GATA3 can be regulated by other transcription factors or self-regulated at the transcriptional level and also degraded through the ubiquitin-proteasome pathway at the protein level (46, 47). The E3 ubiquitin ligases, Mdm2, Fbw7, or FBXW7α, participate in the ubiquitination of GATA3 and promote its proteasomal degradation (48, 49, 50). The deubiquitinase USP21 removes the ubiquitin chain from GATA3 and protects it from degradation in Treg cells (51). Whether and how GATA3 is deubiquitinated in mammary epithelial and tumor cells remains unknown.

In this study, we demonstrated that USP11 binds to and deubiquitinates GATA3, which promotes the transcription of *CDKN1B* and *Esr1*, maintains the luminal characteristics of mammary epithelial and tumor cells, and inhibits their proliferation and migration, thereby suppressing luminal tumor growth and metastasis. Thus, we identified a critical USP11-GATA3 axis that maintains luminal cell fate and restrains luminal cell proliferation and migration in the mammary glands and breast cancers.

## Results

### USP11 suppresses luminal epithelial and tumor cell proliferation, migration, tumorigenesis and metastasis, but promotes basal-like tumor cell proliferation, migration, and tumorigenesis

**Figure 1:**
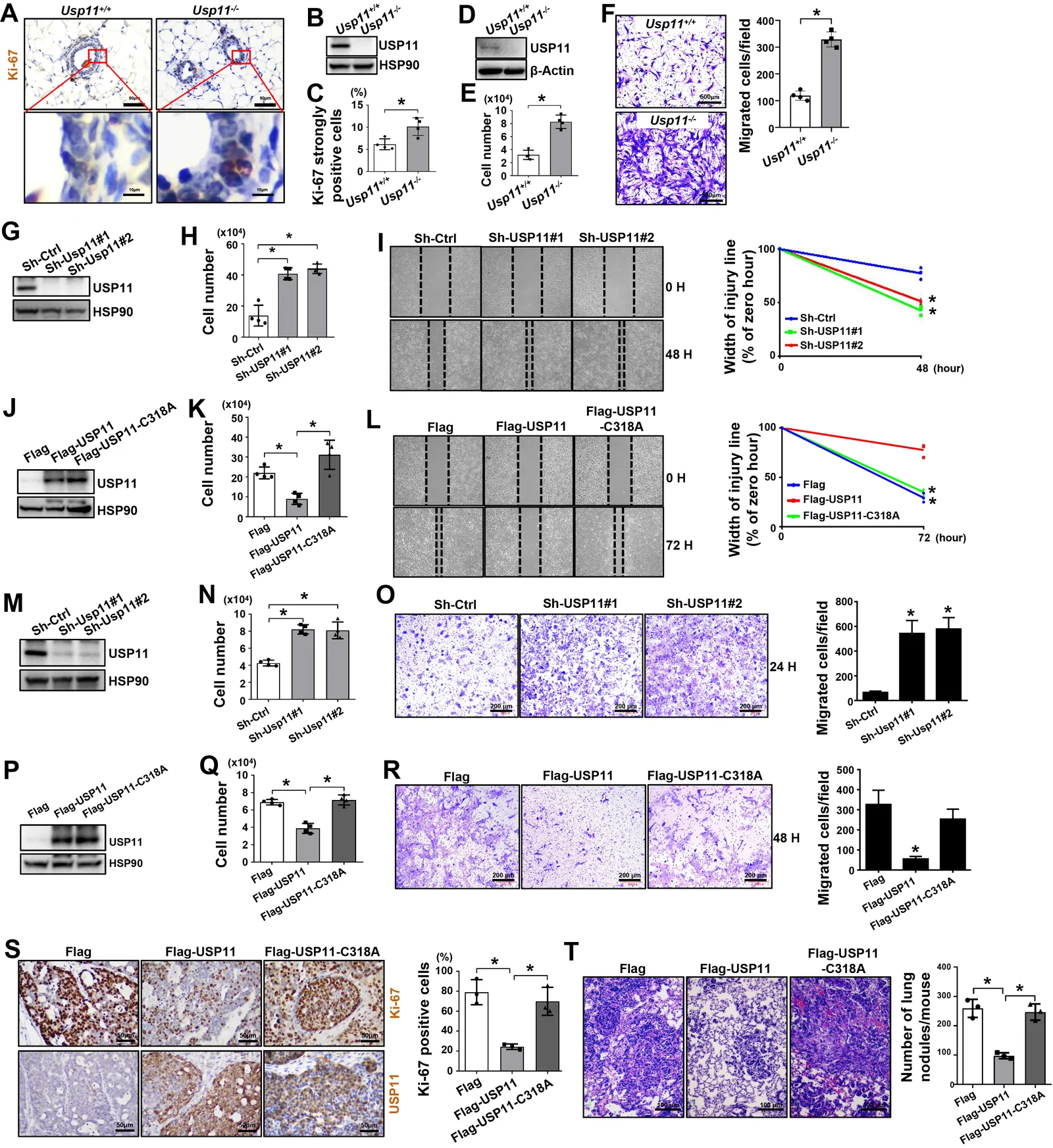
**USP11 negatively regulates the proliferation of luminal-type mammary epithelial and tumor cells.***A* and *B*, representative mammary glands from 5-week-old *Usp11*^*+/+*^ and *Usp11*^*−/−*^ mice were analyzed by IHC staining with an antibody against Ki-67 (*A*), or by western blotting (*B*). HSP90 served as an internal control. *C*, the percentage of Ki-67 strongly positive cells in mammary glands in (*A*) was quantified and plotted in (*C*). n = 6; ∗*p* < 0.05. *D and F*, Primary mammary epithelial cells from 5-week-old *Usp11*^*+/+*^ and *Usp11*^*−/−*^ mice were isolated and maintained in culture medium supplemented with estrogen. *D*, the protein expression of USP11 was examined by western blotting, with β-Actin serving as an internal control. *E*, quantification of the cell number was performed after 2 weeks of reseeding the cells into 6-well plates, starting with 5000 cells for each cell line. n = 4; ∗*p* < 0.05. *F*, (*left*) representative photos from transwell assays of each cell line; (*right*) the number of migrated cells was quantified at 0 and 48 h n = 4; ∗*p* < 0.05. *G* and *L*, T47D cells were infected with Sh-Ctrl, Sh-USP11#1, or Sh-USP11#2 (*G* and *I*) or Flag, Flag-USP11, or Flag-USP11-C318A (*J* and *L*) for 48 h. The cells were then analyzed by western blotting (*G* and *J*), or were seeded into 6-well plates at 5000 cells per well for 2 weeks, and then the cell number was determined. n = 4; ∗*p* < 0.05 (*H* and *K*), or the scratch healing assay was performed (*I* and *L*, *left*, representative photos; *right*, the percentage of gap closure was determined). n = 4; ∗*p* < 0.05. *M* and *R*, PyMT-2095 cells were infected with Sh-Ctrl, Sh-Usp11#1, or Sh-Usp11#2 (*M* and *O*) or Flag, Flag-USP11, or Flag-USP11-C318A (*P* and *R*) for 48 h. The cells were then analyzed by western blotting (*M* and *P*), or were seeded into 6-well plates at 5000 cells per well for 2 weeks, and then the cell number was determined. n = 4; ∗*p* < 0.05 (*N* and *Q*), or the transwell assay was performed (*O* and *R*, *left*, representative photos; *right*, the number of migrated cells was quantified). n = 4; ∗*p* < 0.05. *S*, (*left*) representative IHC staining of Ki-67 and USP11 in tumors generated by PyMT-2095 cells stably overexpressing Flag, Flag-USP11, or Flag-USP11-C318A; (*right*) quantification of Ki-67-positive tumor cells. n = 3; ∗*p* < 0.05. *T*, (*left*) PyMT-2095 cells, infected with Flag, Flag-USP11, or Flag-USP11-C318A, were injected into the tail vein of NCG mice. 22 days post-injection, the mice were sacrificed and the lungs dissected for H&E staining; (*right*) the number of lung nodules was quantified. n = 3; ∗*p* < 0.05. USP11, ubiquitin-specific protease 11.

Recent studies report that that USP11 stimulates the proliferation and migration of MDA-MB231, SUM149, and 4T1 BCs, all of which are basal-like cancer cells (26, 52, 53). However, we previously observed that USP11 reduces the growth of luminal-type tumors generated by *MMTV-PyMT* mammary luminal tumor cells (29). To determine the role of USP11 in controlling the proliferation of normal MECs, we performed immunohistochemistry (IHC) analysis with an antibody against Ki-67, a proliferation indicator (54), in 5-week-old virgin mammary glands. We found that the number of Ki-67-strongly positive MECs in *Usp11*^*−/−*^ mice were significantly higher than that in *Usp11*^*+/+*^ mice, and that most of the Ki-67-strongly positive MECs were inner-layer luminal-type MECs (Fig. 1, *A*–*C*). Whole-mount and histopathological analysis revealed that *Usp11*^*−/−*^ mouse mammary glands developed normally and did not spontaneously form tumors by 12 months of age, as we previously reported (29). We then isolated primary MECs from *Usp11*^*+/+*^ and *Usp11*^*−/−*^ female mice, and cultured them in MM + medium, in which luminal mammary cell fate is well maintained (55). Consistently, *Usp11*^*−/−*^ MECs proliferated faster than *Usp11*^*+/+*^ MECs *in vitro* (Fig. 1, *D* and *E*). In addition, Transwell assay showed that loss of USP11 increased the migration of MECs (Fig. 1*F*). These results indicate that loss of USP11 promotes the proliferation and migration of luminal MECs.

We then examined the role of USP11 in regulating the proliferation and migration of human luminal-type breast cancer T47D and mouse luminal-type mammary tumor PyMT-2095 cells. Knockdown of USP11 by sh-USP11#1 or sh-USP11#2 in T47D cells significantly enhanced the proliferation and migration (Figs. 1, *G*–*I* and S1*A*), whereas overexpression of WT USP11, not of a deubiquitinating enzyme-inactive mutant USP11-C318A, reduced the proliferation and migration in T47D cells (Figs. 1, *J–L* and S1*B*). Similar results were observed in mouse luminal-type mammary tumor PyMT-2095 cells, confirming that USP11 negatively regulated their proliferation and migration (Figs. 1, *M–R* and S1, *C* and *D*).

Taking advantage of mammary tumors generated by Flag-, Flag-USP11-, or Flag-USP11-C318A-stably expressing PyMT-2095 cells (29), we analyzed Ki-67 expression in these tumors. We observed that the number of Ki-67-positive tumor cells in Flag-USP11, but not in Flag-USP11-C318A tumors, was significantly lower than that in Flag tumors (Figs. 1*S* and S1*E*). We then examined the metastatic potential of PyMT-2095 tumor cells overexpressing Flag, Flag-USP11, or Flag-USP11-C318A. We found that WT USP11 overexpression significantly reduced the number of lung metastases compared to Flag-overexpressing controls, whereas USP11-C318A overexpression did not (Fig. 1*T*). These data indicate that WT, but not mutant, USP11 reduces luminal tumor cell proliferation, thereby suppressing tumor growth and inhibits luminal tumor metastasis.

We also investigated the role of USP11 in regulating the proliferation, migration and tumorigenesis of human and mouse basal-like BCs. We found that overexpression of WT USP11, not USP11-C318A, significantly promoted these processes in MDA-MB231 (Fig. S1, *F–K*) and 4T1 (Fig. S1l–*Q*) cells, a finding contrary to the data obtained in luminal tumor cells. However, these results are consistent with previous findings from other groups showing that USP11 promotes mammary tumorigenesis in studies using basal-like BCs (26, 28, 52, 53). Together, these data indicate that USP11 suppresses the proliferation, migration and tumorigenesis of luminal mammary epithelial and tumor cells, but promotes these processes in basal-like mammary tumor cells.

### USP11 positively regulates GATA3, p27, and ERα levels in mammary epithelial and tumor cells

**Figure 2:**
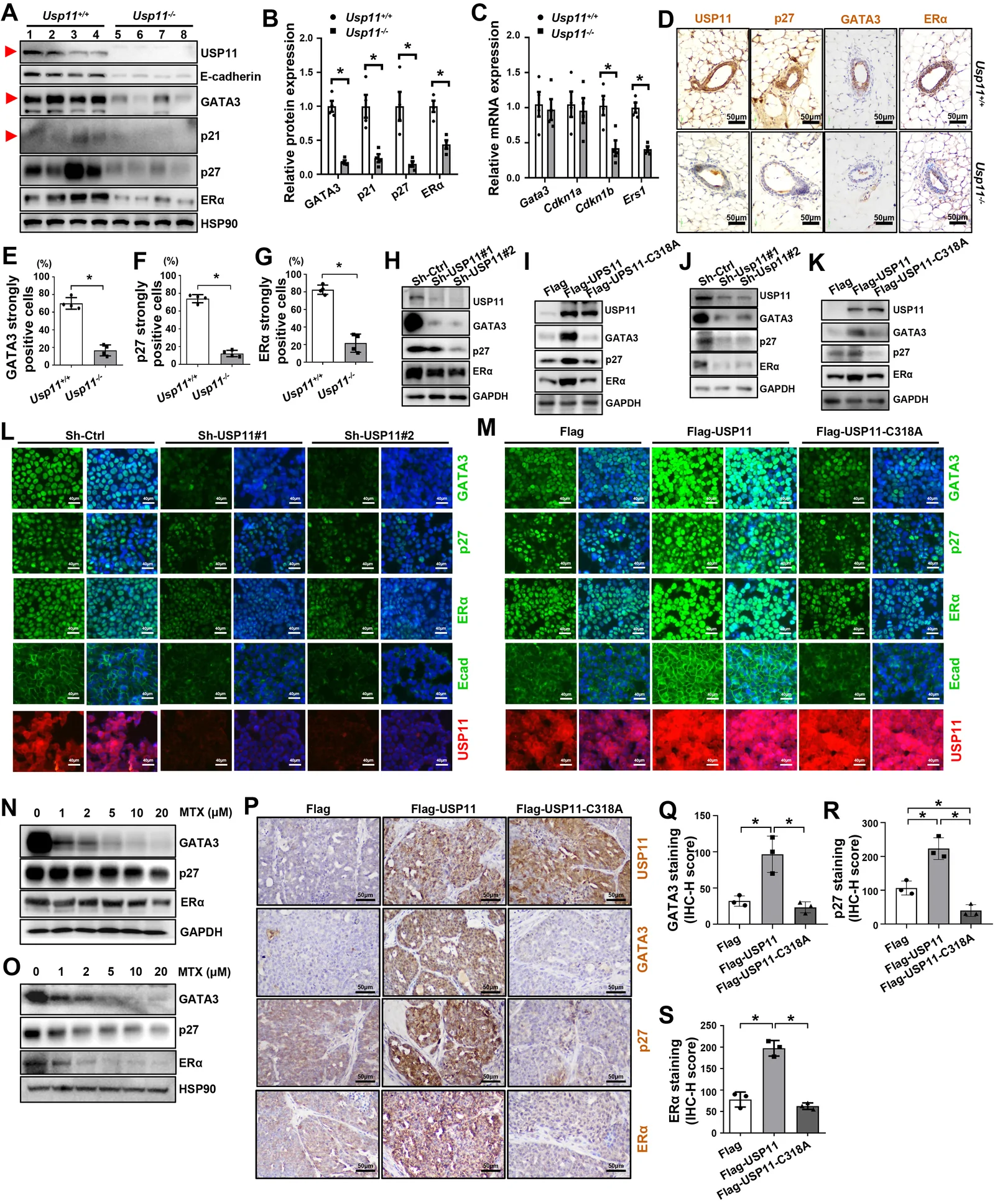
**USP11 maintains GATA3, p27, and ERα expression in mammary epithelial and tumor cells.***A*, mammary glands from 5-week-old *Usp11*^*+/+*^ and *Usp11*^*−/−*^ mice were analyzed, with HSP90 serving as an internal control. *B*, quantification of GATA3, p21, p27, and ERα expression in (*A*) is shown. n = 4; ∗*p* < 0.05. *C*, the mRNA expression of *Gata3*, *Cdkn1a*, *Cdkn1b*, and *Esr1* in mammary glands of 5-week-old *Usp11*^*+/+*^ and *Usp11*^*−/−*^ mice was determined by qRT-PCR and statistically analyzed. n = 4; ∗*p* < 0.05. *D*, representative IHC staining of USP11, GATA3, p27, and ERα in mammary glands of 5-week-old *Usp11*^*+/+*^ and *Usp11*^*−/−*^ mice. *E and G*, quantification of strongly positive cells for GATA3 (*E*), p27 (*F*), and ERα (*G*) in the mammary glands shown in (*D*). n = 4; ∗*p* < 0.05. *H and K*, T47D (*H and I*), and PyMT-2095 (*J and K*) cells were infected with Sh-Ctrl, Sh-USP11#1, or Sh-USP11#2 (*H and J*), or Flag, Flag-USP11, or Flag-USP11-C318A (*I and K*) for 48 h, and then analyzed, with GAPDH serving as an internal control. *L and M*, T47D cells were infected with Sh-Ctrl, Sh-USP11#1, or Sh-USP11#2 (*L*) or Flag, Flag-USP11, or Flag-USP11-C318A (*M*) for 48 h, and then were analyzed by IF, with DAPI serving to show the cell nuclei. *N and O*, T47D (*N*) and PyMT-2095 (*O*) cells were treated with 0, 1, 2, 5, 10, or 20 μm MTX for 24 h, and then GATA3, p27, and ERα were immunoblotted with GAPDH serving as an internal control. *P*, representative IHC staining of USP11, GATA3, p27, and ERα in tumors generated by PyMT-2095 cells stably overexpressing Flag, Flag-USP11, or Flag-USP11-C318A. *Q and S*, IHC-H score of GATA3 (*Q*), p27 (*R*), and ERα (*S*) were analyzed in the tumors in (*P*). n = 3; ∗*p* < 0.05. ER, estrogen receptor; USP11, ubiquitin-specific protease 11.

Given that USP11 maintains the luminal characteristics in MECs and mammary tumor cells (29), and regulates the proliferation and migration of these cells, we examined the expression of E-cad, GATA3, and ERα (three proteins essential for maintaining luminal cell identity), and p21 and p27 (two proteins critical for controlling cell cycle progression) in mouse mammary glands. We found that the protein levels of E-cad, GATA3, p21, p27, and ERα in *Usp11*^*−/−*^ mammary glands were significantly lower than those in *Usp11*^*+/+*^ glands (Fig. 2, *A* and *B*). We previously demonstrated that loss of USP11 reduces E-cad protein, but not mRNA, expression (29), and others have shown that deficiency of USP11 reduces p21 protein expression in cancer cells (18). We, therefore, examined the mRNA levels of *Gata3*, *Cdkn1a* (encoding p21), *Cdkn1b* (encoding p27), and *Esr1* (encoding ERα) in mouse mammary glands. We found that *Cdkn1b* and *Esr1*, not *Gata3* and *Cdkn1a*, mRNA levels in *Usp11*^*−/−*^ mammary glands were significantly lower than those in *Usp11*^*+/+*^ glands (Fig. 2*C*). In addition to confirming the regulation of USP11 on p21, these findings suggest that USP11 positively regulates GATA3 mainly at the protein level, and regulates p27 and ERα at the transcriptional level in MECs. We then focused on the regulation of GATA3, p27, and ERα in luminal MECs *in vivo*. By IHC analysis of 5-week-old virgin mammary glands, we found that the percentages of GATA3-, p27-, or ERα-strongly positive MECs in *Usp11*^*−/−*^ mice were significantly lower than those in *Usp11*^*+/+*^ mice (Fig. 2, *D*–*G*). The increased proliferation of luminal MECs, the decreased number of E-cad-, GATA3-, or ERα-strongly positive luminal MECs, and the reduced level of these proteins in *Usp11*^*−/−*^ breast tissue suggest that USP11-null mammary glands contain a higher proportion of luminal epithelial cells that individually express lower levels of E-cad, GATA3, and ERα. To further validate the regulation of USP11 on GATA3 under physiological conditions, we also checked the expression of GATA3 in the thymus glands, an immune organ known to express high levels of GATA3 (56). We found that GATA3 protein levels in *Usp11*^*−/−*^ thymuses were drastically lower than those in *Usp11*^*+/+*^ thymuses (Fig. S2, *A* and *B*), confirming that loss of USP11 reduces GATA3 expression during organ development *in vivo*.

We then used a lentivirus system to knock down or overexpress USP11 in T47D and PyMT-2095 cells, respectively. We found that knockdown of USP11 reduced the protein expression of GATA3 and p27 in T47D and PyMT-2095 cells compared to that in control cells (Fig. 2, *H* and *J*), whereas overexpression of USP11, but not the USP11-C318A mutant, elevated the protein expression of GATA3 and p27 in T47D and PyMT-2095 cells compared to that in control cells (Fig. 2, *I* and *K*). In addition, IF analysis of T47D cells also confirmed that knockdown of USP11 reduced GATA3 and p27 levels, and overexpression of USP11, not the USP11-C318A mutant, elevated GATA3 and p27 expression compared to that in control cells (Fig. 2, *L* and *M*).

Furthermore, we found that the expression of GATA3, p27, and ERα was inhibited by mitoxantrone (MTX), a USP11 inhibitor, in T47D and PyMT-2095 cells in a dose-dependent manner (Fig. 2, *N* and *O*), supporting the finding that USP11 promotes the expression of these proteins.

**Figure 6:**
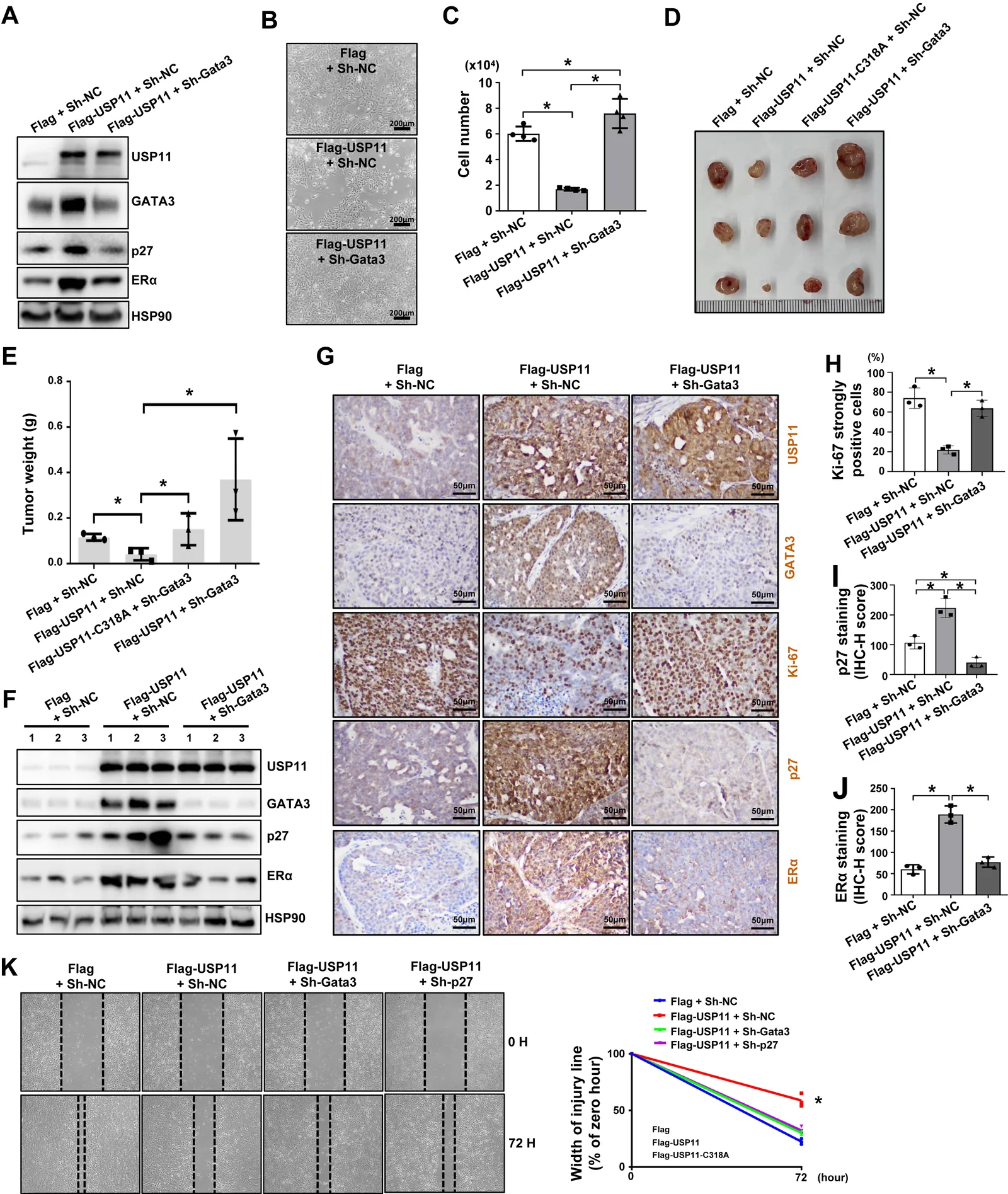
**USP11 maintains the expression of p27 and suppresses luminal mammary tumor cell proliferation through GATA3.***A and C*, PyMT-2095 cells were infected with lentivirus overexpressing Flag or Flag-USP11, along with lentivirus for Sh-Ctrl or Sh-Gata3 knockdown. The expression of USP11, GATA3, p27, and ERα was immunoblotted after 48 h of infection, with HSP90 serving as an internal control (*A*). Cells were reseeded into 6-well plates for 2 weeks, starting with 5000 cells for each cell line. Photos were then taken (*B*) and the cell number was analyzed (*C*). n = 4; ∗*p* < 0.05. *D and J*, PyMT-2095 cells were infected with lentivirus overexpressing Flag, Flag-USP11, or Flag-USP11-C318A, along with lentivirus for Sh-Ctrl or Sh-Gata3 knockdown. Cells were selected with hygromycin and puromycin for 4 days, and then transplanted into the mammary fat pad of NCG mice for 20 days. The appearance of regenerated tumors was photographed (*D*), and the tumor weight was statistically analyzed (*E*). n = 3; ∗*p* <0.05. The expression of USP11, GATA3, p27, and ERα in tumors was examined by immunoblotting, with HSP90 serving as an internal control (*F*). The expression of USP11, GATA3, p27, ERα, and Ki-67 was assessed by IHC staining (*G*). The percentage of Ki-67-positive tumor cells (*H*) and IHC-H scores of p27 (*I*) and ERα (*J*) in *(G*) were statistically analyzed. n = 3; ∗*p* < 0.05. (*K*) (*left*) representative photos from the scratch healing assay of PyMT-2095 cells infected with Flag, Flag-USP11, Flag-USP11 plus sh-GATA3, Flag-USP11 plus sh-p27; (*right*) the gap closure in percent was estimated at 0 and 72 h n = 3; ∗*p* < 0.05. ER, estrogen receptor; USP11, ubiquitin-specific protease 11.

We performed IHC analysis on mammary tumors generated by Flag-, Flag-USP11-, or Flag-USP11-C318A-stably expressing PyMT-2095 cells (see Fig. 6*D*, and reference in QT, JBC, 2024 for details). We found that overexpression of USP11, but not the USP11-C318A mutant, significantly increased the expression of GATA3, p27, and ERα in tumor cells when compared to that in control tumors (Figs. 2, *P*–*S* and S2, *F* and *G*).

To determine whether a feedback regulation exists between GATA3 and USP11, we knocked down GATA3 in T47D cells and found that deficiency of GATA3 reduced the mRNA expression of *CDKN1B*, as expected, but not that of *USP11* (Fig. S2*C*). Overexpression of GATA3 in T47D and *Gata3*^*+/−*^ mammary tumor cells did not alter the protein expression of USP11 (Fig. S2, *D* and *E*). These data suggest that GATA3 does not regulate USP11 in BCs.

In summary, these data demonstrate that USP11 positively regulates GATA3, p27, and ERα expressions in mammary epithelial and tumor cells dependent on its deubiquitinase activity.

### The expression of USP11, GATA3, p27, and ERα is positively correlated with one another in breast cancer

**Figure 3:**
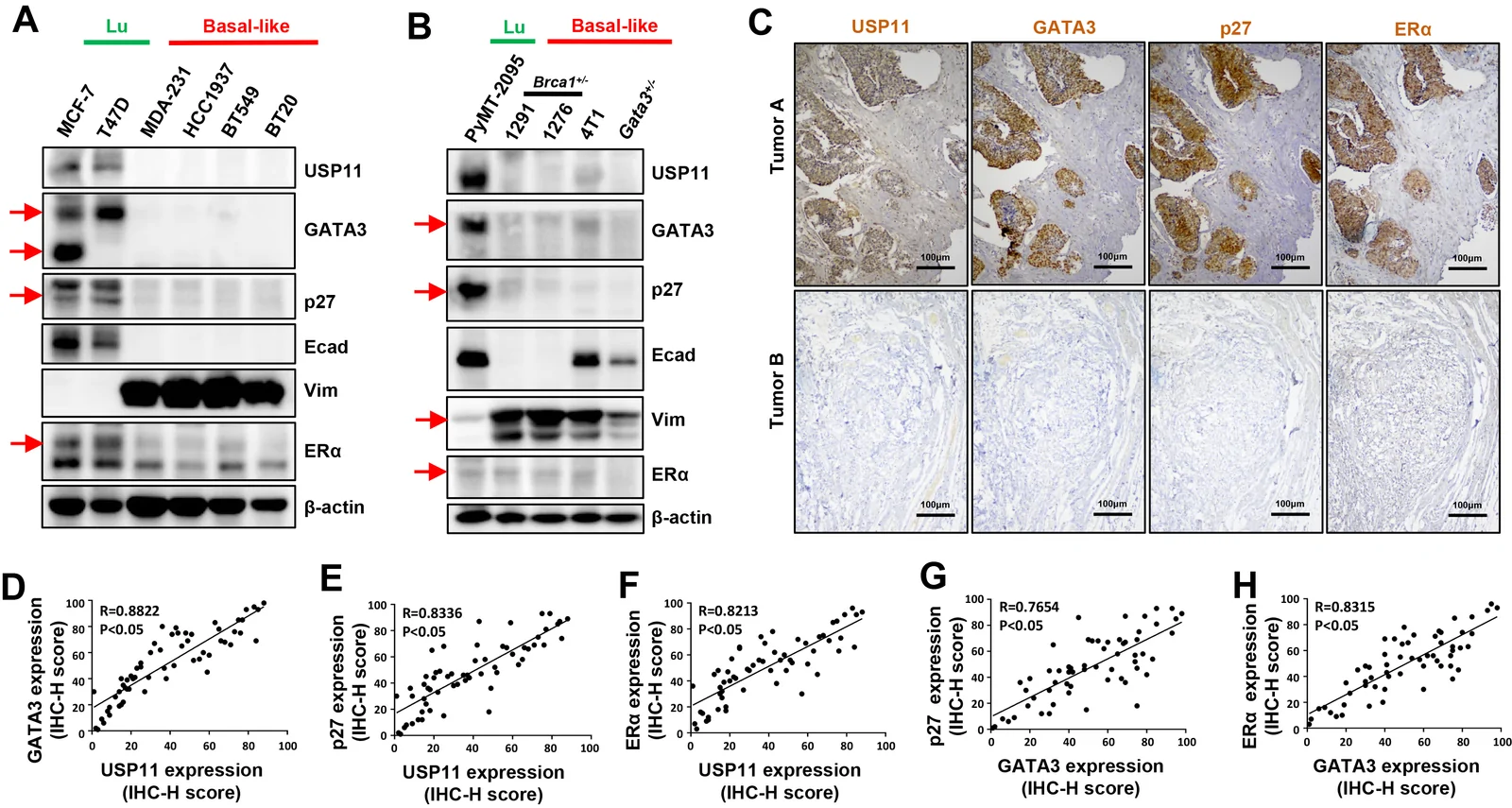
**Expressions of USP11, GATA3, p27, and ERα in BCs and tumors.***A*, the expression of USP11, GATA3, p27, E-cad, Vim, and ERα was examined by immunoblotting in human MCF-7, T47D, MDA-MB231, HCC1937, BT549, and BT20 BC cell lines (*A*), as well as in mouse PyMT-2095, *Brca1*^*+/−*^ (1276 & 1291), 4T1, and *Gata3*^*+/−*^ mammary tumor cell lines (*B*), with HSP90 serving as an internal control. Lu, Luminal. *C*, representative IHC staining of USP11, GATA3, p27, and ERα in serial sections of human breast tumors. *D and H*, the expression correlation analysis of USP11 & GATA3 (*D*), USP11 & p27 (*E*), USP11 & ERα (*F*), GATA3 & p27 (*G*), and GATA3 & ERα (*H*) protein levels for human breast cancer samples from (*C*) was graphed; n = 60. BC, breast cancer; ER, estrogen receptor; USP11, ubiquitin-specific protease 11.

To determine the expression correlation among USP11, GATA3, p27, and ERα, we examined six human and five mouse mammary tumor cell lines. We found that the expression of USP11 was positively correlated with that of the luminal markers GATA3, E-cad, and ERα, as well as the cell cycle inhibitor p27, whereas the expression of USP11 was negatively correlated with that of the mesenchymal marker Vim (Fig. 3, *A* and *B*). Due to the heterogeneity of some mouse basal-like tumor cell lines including 4T1 and *Gata3*^*+/−*^ (7, 55), E-cad was also detected in these cells (Fig. 3*B*).

We next conducted IHC analysis using antibodies against USP11, GATA3, p27, and ERα on serial human breast tumor sections. The tumors stained with each antibody were visualized by microscopy and scored (Fig. 3*C*). Of the 60 tumors analyzed, the expression correlations between USP11 & GATA3, USP11 & p27, USP11 & ERα, GATA3 & p27, and GATA3 & ERα were subsequently determined. We found that, in general, tumors exhibiting USP11 positive staining were accompanied by GATA3, p27, and ERα positive staining, while tumors with USP11 weak or negative staining were accompanied by GATA3, p27, and ERα weak or negative staining (Fig. 3*C*). H-score analyses revealed that the expressions of USP11 & GATA3, USP11 & p27, USP11 & ERα, GATA3 & p27, and GATA3 & ERα were significantly (all *p* < 0.05) and positively (all R >0.3) correlated with each other (Fig. 3, *D*–*H*).

Taken together, these results indicate that expression of USP11, GATA3, p27, and ERα is positively correlated with each other in human breast tumors and cells, further supporting that USP11 maintains the expression of GATA3, p27, and ERα in human BCs.

### USP11 binds to and deubiquitinates GATA3

**Figure 4:**
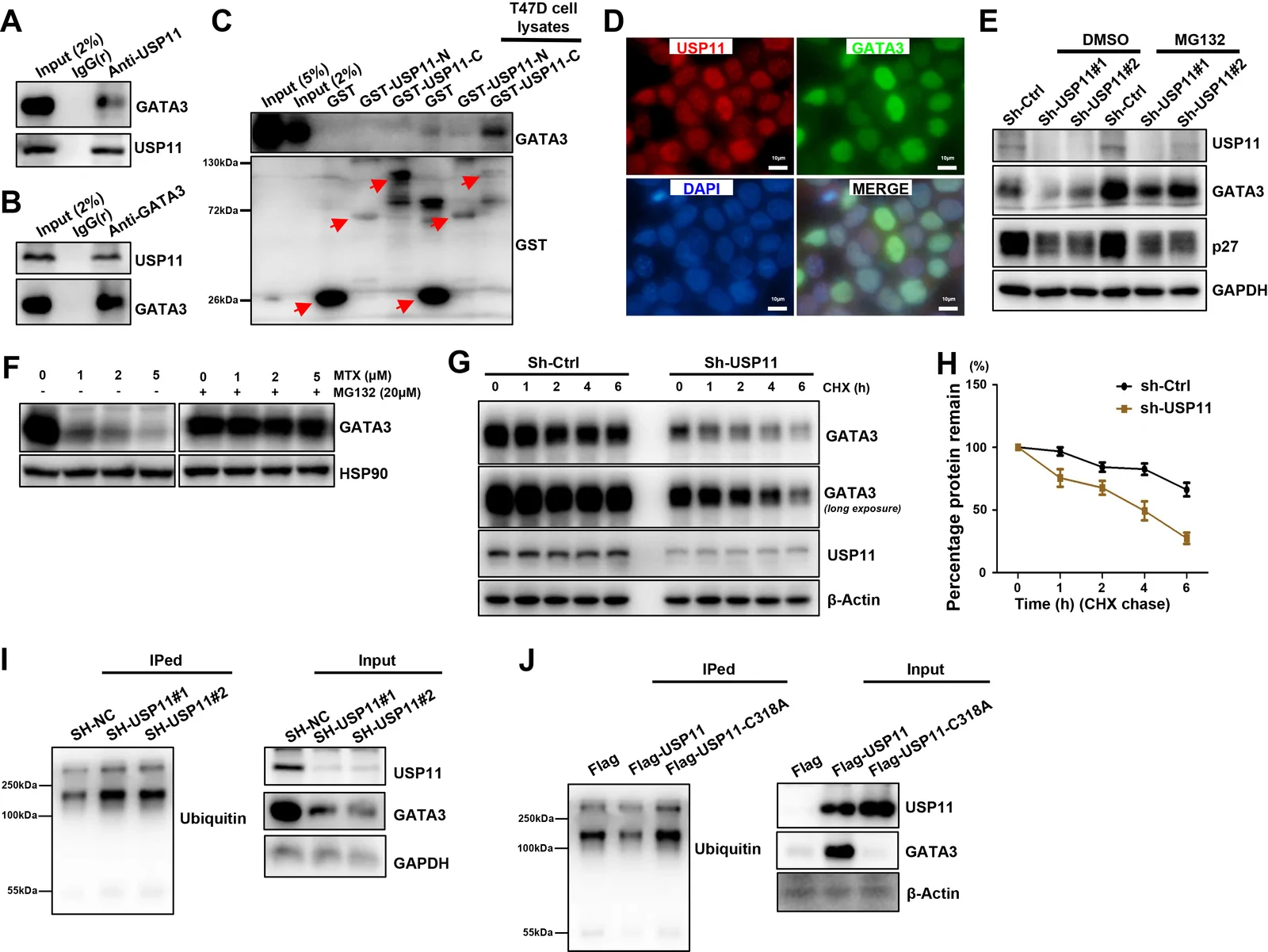
**USP11 is a deubiquitinase for GATA3.***A* and *B*, T47D cell lysates were immunoprecipitated (IPed) with USP11 or GATA3 antibody or control IgG, and the expression of GATA3 and USP11 in input and IPed samples was examined by immunoblotting. *C*, The purified GST, GST-tagged N-terminal fragment of USP11 (GST-USP11-N), and GST-tagged C-terminal fragment of USP11 (GST-USP11-C) were applied to interact with GATA3 from T47D cell lysates. The eluted proteins were then immunoblotted with GST or GATA3 antibodies. *Red arrows* indicate the bands corresponding to GST, GST-USP11-N, and GST-USP11-C. *D*, representative immunofluorescent staining of USP11 (*red*) and GATA3 (*green*) in T47D cells, with DAPI serving to show the cell nuclei. *E*, T47D cells were infected with Sh-Ctrl, Sh-USP11#1, or Sh-USP11#2 lentivirus for 48 h and then treated with 20 μM MG132 or DMSO for an additional 6 h. The expression of USP11, GATA3, and p27 was examined by immunoblotting, with GAPDH serving as an internal control. *F*, T47D cells were treated with MTX along with MG132 or DMSO for 24 h, and then GATA3 was immunoblotted, with HSP90 serving as an internal control. *G and H*, T47D cells were infected with Sh-Ctrl, Sh-USP11#1, or Sh-USP11#2 lentivirus for 48 h and then treated with 3 μM cycloheximide (CHX) for additional 0, 1, 2, 4, or 6 h. The expression of USP11, GATA3, and p27 was examined by immunoblotting (*G*) and quantified (*H*), with β-Actin serving as an internal control. *I* and *J*, T47D cells were infected with Sh-Ctrl, Sh-USP11#1, or Sh-USP11#2 knockdown (*I*) or Flag, Flag-USP11, or Flag-USP11-C318A overexpression (*J*) lentivirus for 48 h, and cell lysates were then IPed with GATA3 antibody. The ubiquitinated GATA3 level in IPed samples and the USP11 and GATA3 levels in input samples were immunoblotted, with GAPDH or β-Actin serving as internal control for input samples. USP11, ubiquitin-specific protease 11.

As a deubiquitinase, USP11 functions in cellular processes mainly by deubiquitinating its substrates. Since USP11 regulates the protein expression of GATA3, p27, and ERα, we investigated whether it deubiquitinates any of these proteins. We performed immunoprecipitation assays in T47D cell lysates using anti-USP11 or anti-GATA3 antibodies to pull down USP11 or GATA3 protein, respectively. By immunoblotting, we found that USP11 was immunoprecipitated by its antibody, and that GATA3 was co-immunoprecipitated with USP11 in the immunoprecipitated sample (Fig. 4*A*). Reciprocal immunoprecipitation revealed that USP11 was co-immunoprecipitated when the GATA3 antibody was used for IP (Fig. 4*B*). However, we did not detect a specific interaction between USP11 & p27 or between USP11 & ERα in T47D cell lysates (Fig. S3*A*).

To clarify which domain of USP11 mediates its interaction with GATA3, we conducted a GST pull-down assay using recombinant GST-USP11-N and GST-USP11-C proteins, along with GST alone as a control, incubated with T47D cell lysates containing endogenous GATA3 protein. We found that only GST-USP11-C, but not GST-USP11-N or GST alone, could pull down GATA3 *in vitro* (Fig. 4*C*). To further determine where USP11 and GATA3 interact with each other in BCs, we performed immunofluorescence staining for USP11 and GATA3 in T47D and PyMT-2095 cells. We found that USP11 and GATA3 staining signals co-localized in the nuclei of T47D and PyMT-2095 cells (Figs. 4*D*, S3*B*). These results suggest that USP11 interacts and co-localizes with GATA3 in the nucleus.

To investigate whether USP11 regulates the stabilization of GATA3, we treated T47D cells with MG132, a proteasome inhibitor that prevents the degradation of ubiquitinated proteins. We found that MG132 drastically reversed the reduction of GATA3, but not that of p27, in USP11-depleted cells relative to that in control cells (Fig. 4*E*). Additionally, MG132 also prevented USP11 inhibitor MTX-induced GATA3 degradation in T47D cells (Fig. 4*F*). These data indicate that deficiency of USP11 promotes proteasome-mediated degradation of GATA3, but not p27. We treated T47D cells with cycloheximide (CHX), a drug that inhibits *de novo* protein synthesis, and found that after a 6-h treatment, 66.2% of GATA3 remained in control cells, whereas only 27.4% of GATA3 remained in USP11-depleted cells (Fig. 4, *G* and *H*), supporting that USP11 knockdown accelerated the degradation of GATA3.

Next, we assessed the levels of the ubiquitinated GATA3 in T47D cells. We found that knockdown of USP11 increased the level of ubiquitinated GATA3 compared to that in the control (Fig. 4*I*). Consistently, overexpression of WT USP11, but not the USP11-C318A mutant, reduced the level of ubiquitinated GATA3 compared to that in the control (Fig. 4*J*). These results confirm that USP11 deubiquitinates GATA3.

### GATA3 binds to and stimulates CDKN1B promoter activity, and USP11 stimulates CDKN1B transcription through GATA3

**Figure 5:**
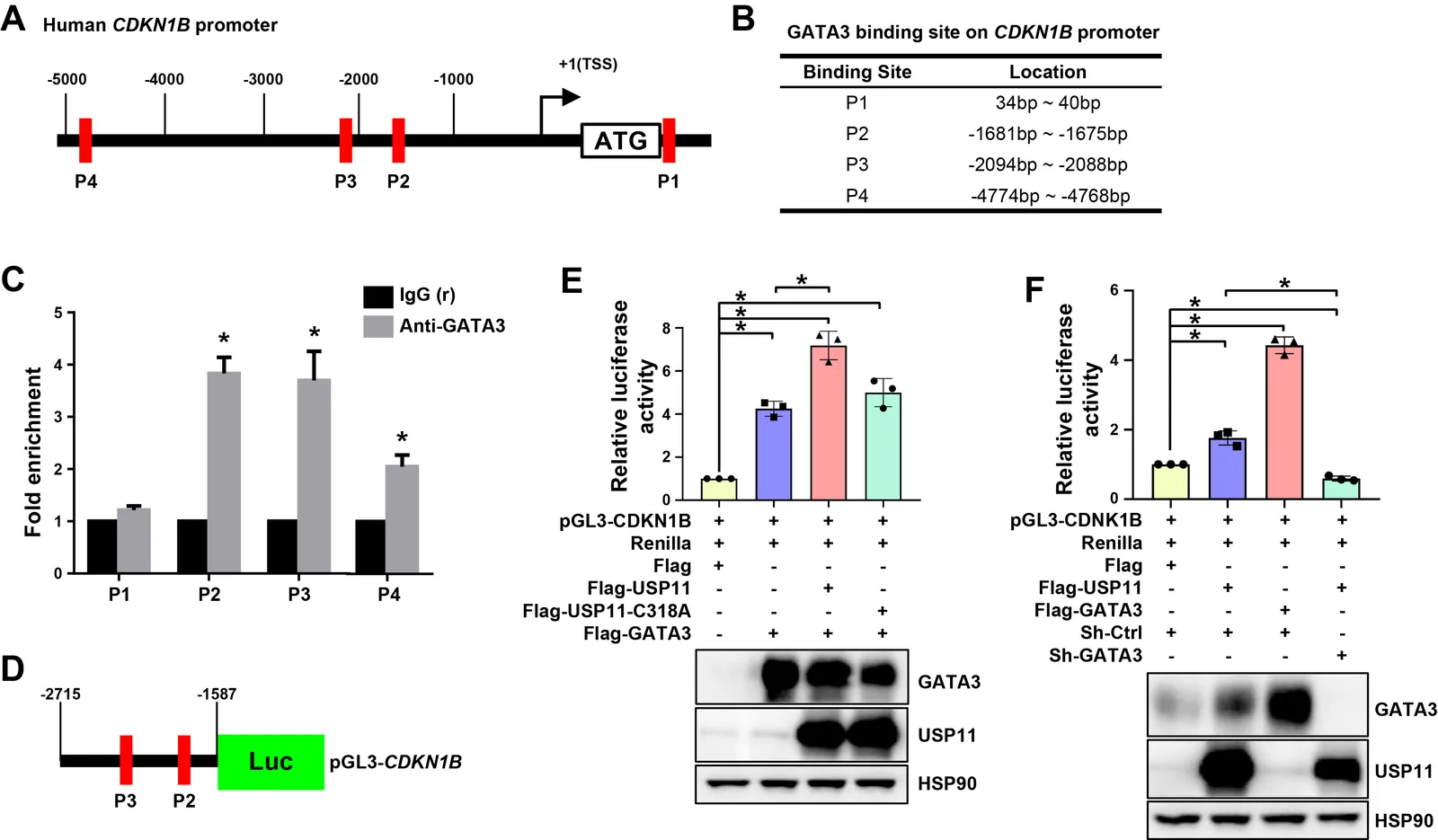
**USP11 collaborates with GATA3 to promote the *CDKN1B* promoter activity.***A*, a schematic diagram displays the putative GATA3 binding sites within the human *CDKN1B* gene near the transcriptional start site (TSS). *B*, location of the GATA3 binding sites on the human *CDKN1B* promoter. *C*, four endogenous GATA3 binding sites within the *CDKN1B* gene near the TSS in T47D cells were analyzed by ChIP assay and quantified by qRT-PCR. *D*, a schematic diagram displays the structure of the constructed pGL3-*CDKN1B* vector, including the P2 and P3 GATA3 binding sites within the *CDKN1B* gene. *E* and *F*, HEK293T cells were transfected with pGL3-*CDKN1B* and Renilla, together with PLVX-Flag, PLVX-Flag-USP11, PLVX-Flag-USP11-C318A, PLVX-Flag-GATA3, PLKO-Sh-Ctrl, or PLKO-Sh-GATA3 plasmids for 48 h. Then, cell lysates were collected and analyzed for pGL3-*CDKN1B* luciferase activity and immunoblotted for GATA3 and USP11, with HSP90 serving as an internal control. n = 3; ∗*p* < 0.05. USP11, ubiquitin-specific protease 11.

To determine how GATA3 regulates *CDKN1B* transcription, we performed bioinformatic analysis of the human *CDKN1B* promoter sequence. We discovered that there exist, at least, four GATA3 binding sites around the TSS (+1), namely P1 (+34 bp to +40 bp), P2 (−1681 bp to −1675 bp), P3 (−2094 bp to −2088 bp), and P4 (−4774 bp to −4768 bp) (Fig. 5, *A* and *B*). Chromatin-immunoprecipitation analysis using the anti-GATA3 antibody revealed that P2, P3, and P4, the former two in particular, exhibited significant GATA3 binding activity (Fig. 5*C*).

We cloned a 1129-bp fragment of the *CDKN1B* promoter spanning from −2715 bp to −1587 bp and covering the P2 and P3 GATA3 binding sites. We then fused this fragment into the pGL3-basic vector as pGL3-*CDKN1B* for conducting the promoter-luciferase reporter assay (Fig. 5*D*). We co-transfected pGL3-*CDKN1B* and Renilla plasmids with Flag, Flag-USP11, Flag-USP11-C318A, or Flag-GATA3 plasmids into HEK293T cells. We found that overexpression of Flag-GATA3 increased the activity of the pGL3-*CDKN1B* promoter by 4.25-fold, and this increased activity was further enhanced by co-overexpression of Flag-USP11 to 7.19-fold compared to that of Flag (Fig. 5*E*). Unlike Flag-USP11 overexpression, Flag-USP11-C318A overexpression did not further increase the promoter activity (Fig. 5*E*). These data suggest that GATA3 stimulates *CDKN1B* promoter activity and that USP11 enhances *CDKN1B* promoter activity through increasing GATA3 stability.

In addition, we co-transfected pGL3-*CDKN1B* and Renilla plasmids with Flag, Flag-USP11, or Flag-GATA3 plasmids, along with Sh-Control (Ctrl) or Sh-GATA3 knockdown plasmids, into HEK293T cells. We observed that, compared with the Flag overexpression condition in the Sh-Ctrl group, the presence of Flag-USP11 or Flag-GATA3 elevated the activity of the pGL3-*CDKN1B* promoter by 1.76-fold and 4.43-fold, respectively. However, compared to the Flag or Flag-USP11 overexpression conditions in Sh-Ctrl groups, co-transfection of Flag-USP11 with Sh-GATA3 significantly reduced pGL3-*CDKN1B* promoter activity (Fig. 5*F*). Consistent with these results, endogenous GATA3 protein levels were increased by Flag-USP11 and drastically decreased by Sh-GATA3. These data indicate that USP11 stimulates *CDKN1B* promoter activity in a GATA3-dependent manner.

Together, these results demonstrate that GATA3 binds to and transcriptionally activates *CDKN1B* expression, and that USP11 stimulates *CDKN1B* transcription in a GATA3-dependent manner.

### Deficiency of GATA3 reverses the proliferative and migratory inhibition induced by USP11 in luminal-type mammary tumor cells

To investigate whether GATA3 mediates the regulation of USP11 on proliferation and migration in BCs, we infected PyMT-2095 cells with lentivirus expressing Flag or Flag-USP11, along with Sh-Ctrl or Sh-Gata3 lentivirus. We confirmed that overexpression of USP11 up-regulated GATA3, p27, and ERα protein levels (Fig. 6*A*). Notably, the up-regulation of p27 and ERα by USP11 was abolished when GATA3 was knocked down (Fig. 6*A*). After re-seeding these cells for 2 weeks, we found that the number of cells in the Flag-USP11 group was significantly less than that in the Flag group or the Flag-USP11 plus Sh-GATA3 group, indicating that knockdown of GATA3 significantly reversed the proliferative inhibition induced by USP11 overexpression (Fig. 6, *B* and *C*).

**Figure 7:**
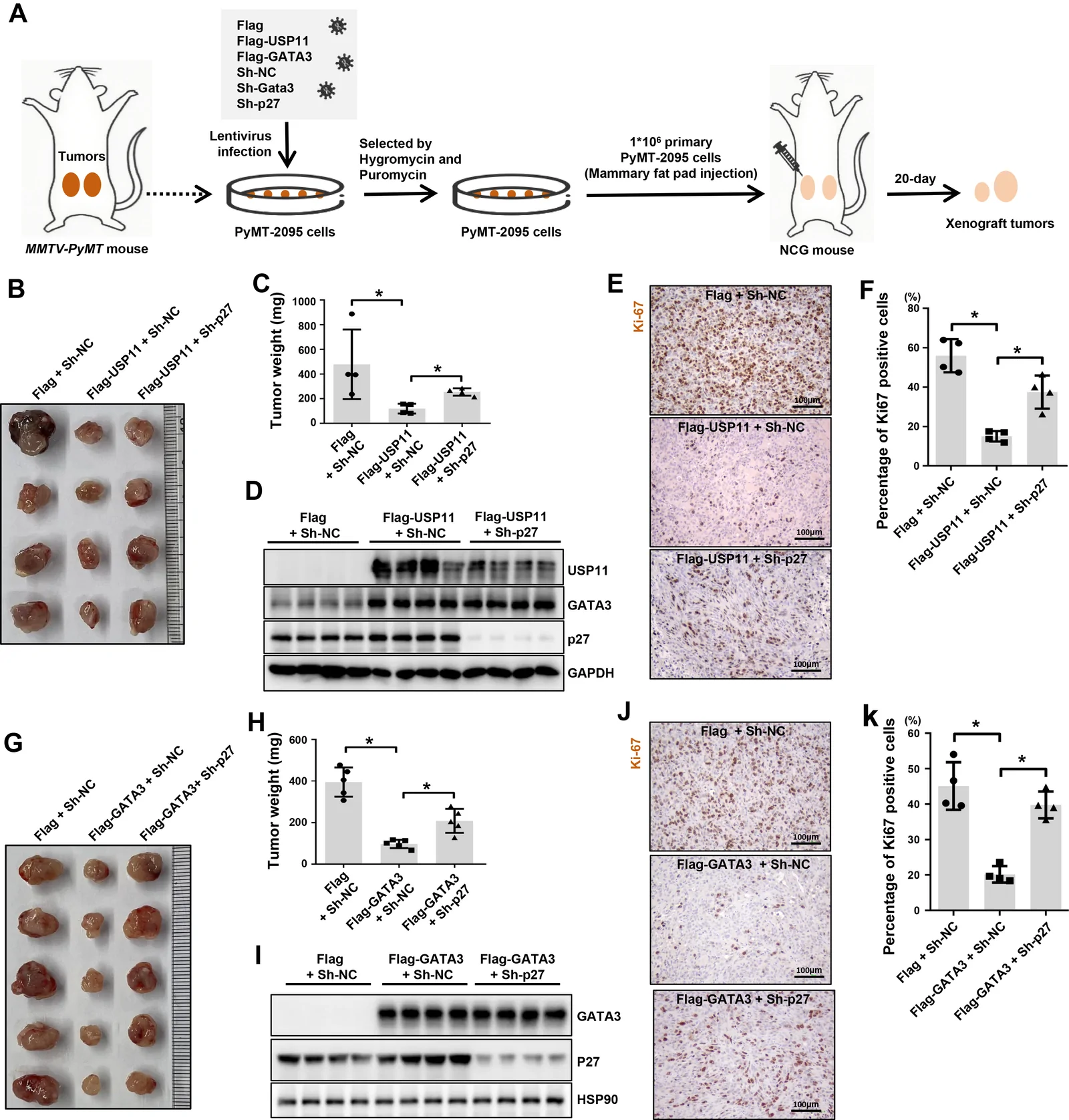
**USP11-GATA3 axis inhibits luminal mammary tumor growth partially dependent on p27.***A*, xenograft models of PyMT-2095 cells infected with Flag, Flag-USP11, or Flag-GATA3 alone or combined with Sh-NC, Sh-Gata3, or Sh-p27 lentivirus were transplanted into the NCG mouse mammary fat pad. *B* and *K*, PyMT-2095 cells infected with various lentiviruses were transplanted into the mammary fat pat of NCG mice. Twenty days post-transplantation, the appearance of regenerated tumors was photographed (*B* and *G*); the tumor weight was analyzed (*C* and *H*); the expression of the indicated genes in the tumors was analyzed by Western blot (*D* and *I*) and IHC (*E* and *J*). n = 4 for (*C*); n = 5 for (*H*). The percentage of Ki-67-positive cells in (*E* and *J*) was quantified (*F* and *K*). n = 4 for each group; ∗*p* < 0.05. USP11, ubiquitin-specific protease 11.

We then generated stable PyMT-2095 cell lines expressing Flag, Flag-USP11, Flag-USP11-C318A, and Flag-USP11 plus Sh-Gata3, and transplanted them into the mammary fat pads of NCG mice (Fig. 7*A*). We found that, consistent with our previous findings, tumors generated by Flag-USP11, but not Flag-USP11-C318A, were significantly smaller and lighter than those generated by Flag (Fig. 6, *D* and *E*). Tumors generated by Flag-USP11 plus Sh-Gata3 were significantly larger and heavier than those generated by Flag-USP11 alone (Fig. 6, *D* and *E*). Western blot analysis revealed that, similar to the *in vitro* cultured cells (Fig. 6*A*), tumors generated by Flag-USP11 expressed higher levels of GATA3, p27, and ERα than those generated by Flag, but lower levels of p27 and ERα than those generated by Flag-USP11 plus Sh-Gata3 (Fig. 6*F*). These results indicate that overexpression of USP11 inhibits luminal tumor growth with induction of GATA3, p27 and ERα, which is reversed by knockdown of GATA3. Next, we performed IHC analysis on those tumors. We confirmed the expression of USP11 and GATA3 in the tumors as expected, and observed that p27 and ERα levels were increased and Ki-67-positive cells were reduced in Flag-USP11 tumors relative to those in Flag tumors (Figs. 6, *G*–J and S4, *A* and *B*). However, p27 and ERα levels were significantly decreased and Ki-67-positive cells were increased in tumors generated by Flag-USP11 plus Sh-Gata3 relative to those generated by Flag-USP11 alone (Fig. 6, *G*–*J*). These data suggest that GATA3 knockdown reverses the USP11 overexpression-induced increase in p27 and ERα as well as the decrease in cell proliferation during luminal-type mammary tumorigenesis.

Finally, we examined the migration of PyMT-2095 cells expressing Flag, Flag-USP11, Flag-USP11 plus Sh-Gata3, or Flag-USP11 plus Sh-p27 using a scratch healing assay. We found that USP11 overexpression again reduced gap closure compared to the control, and that knockdown of GATA3 or p27 reversed the inhibitory effect of USP11 overexpression on cell migration in PyMT-2095 cells (Fig. 6*K*).

In summary, these results indicate that USP11 stabilizes the GATA3 protein, inducing the expression of p27 and ERα, and eventually leading to inhibition of luminal tumor cell proliferation and migration.

### P27 knockdown rescues the proliferative and migratory inhibition induced by USP11 or GATA3 in luminal-type mammary tumor cells

Since p27 was positively regulated by the USP11-GATA3 axis in BCs (Figs. 2 and 6), we then determined whether p27 plays a key role in the USP11-GATA3 axis-mediated tumor suppression. We generated stable PyMT-2095 cell lines expressing Flag, Flag-USP11, Flag-GATA3, Flag-USP11 plus Sh-p27, or Flag-GATA3 plus Sh-p27. We then transplanted these cells into the mammary fat pads of NCG mice (Fig. 7*A*), and found that tumors generated by Flag-USP11 plus Sh-p27 were significantly larger and heavier than those generated by Flag-USP11 alone (Fig. 7, *B* and *C*). IHC analysis confirmed that the former expressed significantly more Ki-67 than the latter (Fig. 7, *E* and *F*). Similarly, we also observed that tumors generated by Flag-GATA3 plus Sh-p27 were significantly larger and heavier, and contained more Ki-67-positive cells than those generated by Flag-GATA3 alone (Fig. 7, *G* and *H*, *J* and *K*). Western blot analysis confirmed the induction of p27 by overexpression of USP11 or GATA3 and the efficient knockdown of p27 in tumors (Fig. 7, *D* and *I*). IHC staining also revealed the efficient knockdown of p27 in tumors (Fig. S5, *A* and *B*).

We also examined the effect of p27 knockdown on cell migration in USP11-overexpressing PyMT-2095 cells using a scratch healing assay. We found that p27 knockdown abolished the inhibitory effect of USP11 on cell migration (Fig. 6*K*).

These data indicate that p27 knockdown significantly reverses the tumor growth inhibition induced by USP11 or GATA3, supporting the conclusion that p27 is a critical downstream target of the USP11-GATA3 axis in suppressing the proliferation of luminal breast cancer cells.

### USP11 overexpression sensitizes luminal BCs to tamoxifen treatment

**Figure 8:**
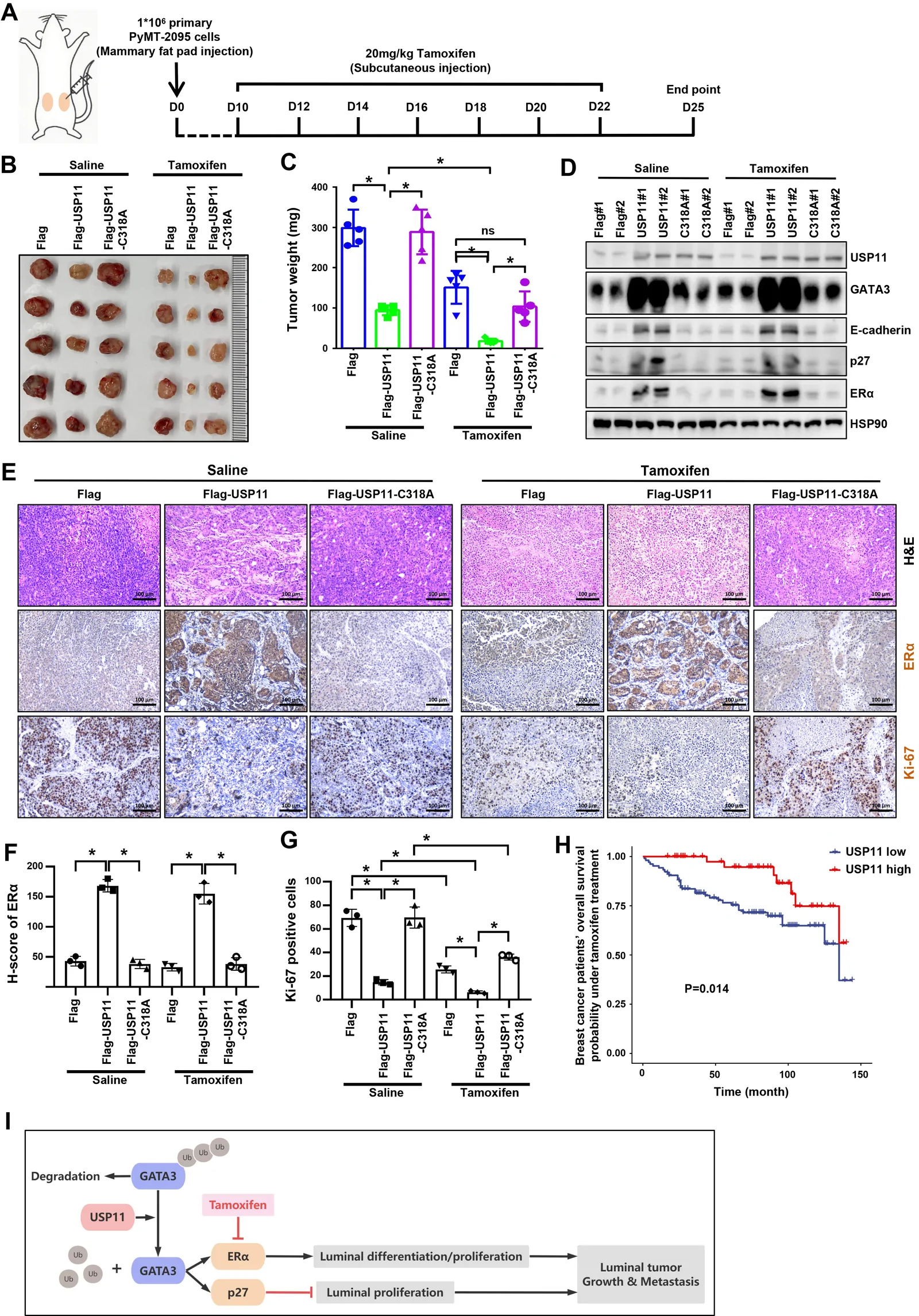
**Overexpression of USP11 promotes tamoxifen efficacy in mammary tumor treatment.***A*, tamoxifen injection schedule for NCG mice injected with 1 million PyMT-2095 cells overexpressing Flag, Flag-USP11, or Flag-USP11-C318A. *B* and *C*, twenty-five days post-transplantation, mice were sacrificed, photographed for tumor appearance (*B*), and analyzed for tumor weight (*C*). n = 5; ∗*p* < 0.05. *D*, protein expression of USP11, GATA3, p27, ERα, and E-cad in tumors in (*B*) was examined by western blotting. *E and G*, The expression of ERα and Ki-67 in tumors in (*B*) was analyzed by IHC staining (*E*) and quantified *F and G*,. n = 3; ∗*p* < 0.05. *H*, Kaplan–Meier analysis of overall survival in breast cancer patients treated with tamoxifen, based on the public GEO dataset GSE58644. Patients were stratified by high *versus* low USP11 expression levels using the median as the cutoff. Higher USP11 expression was associated with significantly better overall survival (log-rank test). Follow-up data were available up to 150 months from initiation of tamoxifen treatment. *I*, A model for the USP11-GATA3 signaling axis in regulating proliferation and migration in luminal breast cancer cells. BCs, breast cancer cells; ER, estrogen receptor; USP11, ubiquitin-specific protease 11.

Our findings that USP11 promotes the expression of GATA3 and ERα (Figs. 2 and 3) in luminal mammary tumor cells allow us to hypothesize that USP11 overexpression can sensitize luminal BCs to tamoxifen treatment (37, 57). We injected tamoxifen into mice bearing tumors generated by PyMT-2095 cells stably overexpressing Flag, Flag-USP11, or Flag-USP11-C318A (Fig. 8*A*). We found that tumors expressing Flag-USP11, but not Flag-USP11-C318A, were significantly smaller and lighter than those expressing Flag in both saline- and tamoxifen-treated groups (Fig. 8, *B* and *C*). Tumors expressing Flag, Flag-USP11, or Flag-USP11-C318A and treated with tamoxifen were clearly lighter than their counterparts treated with saline. Notably, compared with saline treatment, tamoxifen reduced tumor weight more substantially in Flag-USP11-expressing tumors (0.1882-fold) than in Flag-expressing tumors (0.5067-fold) or Flag-USP11-C318A-expressing tumors (0.3585-fold) (Fig. 8*C*). These data suggest that overexpression of USP11 enhances the efficacy of tamoxifen treatment for luminal breast cancer. Western blot analysis of those xenograft tumors confirmed that overexpression of Flag-USP11, but not Flag-USP11-C318A, increased the expression of GATA3, p27, ERα, and E-cad in both saline- and tamoxifen-treated tumors (Fig. 8*D*). IHC analysis of these tumors further confirmed the strong induction of ERα in Flag-USP11, but not Flag-USP11-C318A, tumors (Fig. 8, *E* and *F*). We also examined the proliferative status of those tumors by IHC staining of Ki-67. We found that the percentages of Ki-67-positive cells in tamoxifen-treated Flag, Flag-USP11, and Flag-USP11-C318A tumors were significantly lower than those in saline-treated tumors, respectively (Fig. 8, *E* and *G*). Finally, to determine whether USP11 expression correlates with the responsiveness of human breast cancers to tamoxifen, we analyzed a publicly available gene expression dataset (GSE58644; Tofigh *et al.*, 2014) (58) from breast cancer patients treated with tamoxifen. The results showed that higher USP11 expression levels were associated with significantly better overall survival measured from the initiation of tamoxifen treatment (Fig. 8*H*), confirming our findings from the mouse model.

In summary, these results indicate that USP11 deubiquitinates GATA3 in luminal mammary tumor cells, promoting the expression of ERα, which sensitizes them to tamoxifen treatment (Fig. 8*I*).

## Discussion

In this study, we demonstrate that USP11 inhibits the proliferation and migration of luminal-type mammary epithelial and tumor cells, but not basal-like tumor cells. USP11 suppresses luminal tumorigenesis and metastasis but promotes basal-like tumorigenesis. USP11 positively regulates GATA3, p27, and ERα in mammary epithelial and tumor cells in a manner dependent on its deubiquitinase activity. USP11 deubiquitinates GATA3, which in turn binds to and transcriptionally activates the *CDKN1B* gene. Consequently, USP11 stimulates *CDKN1B* and *ESR1* transcription in a GATA3-dependent manner. GATA3 knockdown reverses the USP11 overexpression-induced upregulation of p27 and ERα, as well as the suppression of proliferation and migration. Furthermore, p27 knockdown rescues the proliferative inhibition induced by USP11 or GATA3 overexpression in luminal-type mammary tumorigenesis. USP11 overexpression sensitizes luminal tumor cells to tamoxifen treatment. The expression levels of USP11, GATA3, p27, and ERα were positively correlated with each other in human breast cancers and cell lines. Thus, we have identified a critical USP11-GATA3 axis that maintains luminal cell fate and restrains luminal cell proliferation and migration in mammary glands and breast cancers.

The role of USP11 in regulating mammary cell proliferation and migration is quite puzzling. It has been reported that USP11 promotes the proliferation and migration of basal-like BCs, such as MDA-MB-231 and 4T1, by binding to and deubiquitinating XIAP, cytoplasmic p21, or PGAM5, or by enhancing the TGF-β signaling pathway (26, 28, 52, 53). Interestingly, USP11 may also inhibit the proliferation and migration of prostate cancer cells and mouse embryonic fibroblasts by deubiquitinating PTEN(22). However, the effect of USP11 on proliferation and migration in luminal BCs remains poorly understood, despite the fact that luminal breast cancer accounts for approximately 80% of all breast cancer cases and USP11 plays a critical role in maintaining luminal cell fate (29). In this study, we report for the first time a unique mechanism by which USP11 regulates the proliferation and migration of luminal BCs, which is distinct from that observed in basal-like cancer cells. We demonstrated that USP11 promotes the transcriptional expression of *CDKN1B* and *ESR1* by deubiquitinating the transcription factor GATA3, thereby inhibiting the proliferation of luminal BCs. The discovery of the USP11-GATA3 axis in mammary luminal cells indicates a unique role of USP11 in maintaining luminal cell fate by stabilizing GATA3, which in turn restrains luminal cell proliferation and migration. These findings strongly suggest that the USP11-GATA3 axis is a specific feature of luminal breast cancer that could serve as a biomarker for this disease.

Notably, we also utilized human and mouse basal-like BCs, MDA-MB-231 and 4T1, which express no or low levels of GATA3(35, 59), and revealed that USP11 did not suppress, but rather promoted, their proliferation and migration during tumorigenesis. These results not only confirm the published findings that USP11 promotes the proliferation and migration of basal-like BCs, but also support our discovery that USP11 suppresses the proliferation and migration of luminal BCs in a GATA3-dependent manner. Although the mechanisms by which USP11 promotes the proliferation and migration of basal-like BCs remain to be determined, data from this study and other publications suggest that USP11 regulates the proliferation and migration of basal-like BCs independently of GATA3, since these cells express no or low levels of GATA3, a factor that suppresses basal-like tumor cell proliferation and migration (7, 26, 52, 53, 59).

GATA3 is a central orchestrator of the luminal phenotype in mammary epithelial and tumor cells, driving the expression of luminal-specific genes such as ERα and E-cad while repressing basal-like and mesenchymal genes, including SNAIL, ZEB, and TWIST (35, 36). This dual role of GATA3 not only reinforces luminal identity but also suppresses the emergence of basal-like characteristics. The stability of the GATA3 protein is tightly regulated by post-translational modifications, including the ubiquitin-proteasome pathway. Several E3 ubiquitin ligases, including Mdm2, Fbw7, and FBXW7α, have been identified to target GATA3 for proteasomal degradation, and the deubiquitinase USP21 counteracts this process (48, 49, 50, 51). However, most of these discoveries were achieved *ex vivo*, and apart from the data on FBXW7α in BCs, the other results were obtained in T cells. In this study, we utilized genetic and biochemical approaches and identified USP11 as a novel deubiquitinating enzyme for GATA3 in mammary epithelial and BCs *in vivo*. We demonstrated that USP11 deubiquitinates and stabilizes GATA3, thereby preventing its degradation and maintaining luminal cell fate in the normal mammary gland and breast cancer. We also demonstrated that USP11 promotes ERα expression through the deubiquitination of GATA3. These findings, coupled with our previous discovery that USP11 also deubiquitinates E-cad to sustain luminal characteristics (29), highlight the pivotal role of USP11 in preserving luminal characteristics in normal and cancerous mammary cells. These insights underscore the multifaceted regulatory mechanisms by which USP11 contributes to the maintenance of luminal identity and reveal its potential therapeutic implications in breast cancer.

Given the critical role of the USP11-GATA3 axis in maintaining luminal cell fate in breast cancer, it would be interesting to investigate whether this axis plays similar or context-dependent roles in other malignancies. In prostate cancer, GATA3 inhibits the Akt pathway and acts as a tumor suppressor, while USP11 stabilizes PTEN and also inhibits the Akt pathway to suppress tumorigenesis, suggesting that the USP11-GATA3 axis may function as a tumor-suppressive axis to delay prostate cancer progression (22, 60). In T-cell acute lymphoblastic leukemia, both USP11 and GATA3 function as oncogenes, raising the possibility that USP11 stabilizes GATA3 to promote leukemogenesis (61, 62). Future studies are warranted to examine whether USP11 directly deubiquitinates and stabilizes GATA3 in these contexts, and whether targeting this axis—either by restoring it in prostate cancer or inhibiting it in T-cell acute lymphoblastic leukemia—could offer therapeutic benefits.

Not until recently has the function of GATA3 in regulating cell proliferation been reported. Although deficiency of Gata3 impairs the proliferation of hematopoietic stem cells (HSCs) (63), the discrepant finding that deletion of *Gata3* enhances the self-renewal of HSCs has also been observed (64). We and others have demonstrated that depletion of Gata3 impairs T-cell proliferation (40, 41, 42), but promotes B cell proliferation and B cell lymphomagenesis (42). GATA3 maintains the quiescent state of cochlear supporting cells by regulating p27 (65). The regulation of cell proliferation by GATA3 is mainly mediated through its modulation of cell cycle-related genes. GATA3 binds to and inhibits the transcription of *p15*^*Ink4b*^ in developing thymocytes (66), and negatively regulates *p16*^*Ink4a*^ and *p15*^*Ink4b*^ in choriocarcinoma cells (67). Heterozygous loss of *Gata3* increases the expression of *p16*^*Ink4a*^, *p18*^*Ink4c*^, and *p19*^*Ink4d*^ in CD4^+^CD8^+^ thymocytes (42). Loss of Gata3 reduces the proliferation of mammary luminal epithelial cells (7, 43) and T cells (41, 42) with induction of *p18*^*Ink4c*^. GATA3 binds to Smad4 and then abrogates the binding of Smad4 to the promoter of *CDKN1A* in basal-like MDA-MB231 cells (68), impairing tumor cell progression. GATA3 can bind to the promoter of and positively regulate the transcription of both mouse *Cdkn1b* and *Cdkn1c* genes in the lens (69). While deficiency of Gata3 reduces the proliferation of mammary luminal epithelial cells (7, 43), it promotes mammary tumorigenesis in a p18^Ink4c^-null background (7). Overexpression of GATA3 in basal-like BCs suppresses proliferation (26, 52). How GATA3 regulates mammary luminal tumor cell proliferation remains poorly understood. In this study, we identified the GATA3 binding sites on the human *CDKN1B* gene (encoding p27) promoter, and confirmed the transactivation of *CDKN1B* by GATA3 in luminal BCs. We demonstrated that GATA3, as a downstream target of USP11, inhibits luminal BC proliferation through promoting *CDKN1B* transcription.

ERα is the major driver for mammary gland development and luminal BC growth and metastasis (70, 71). Targeting the transcriptional potency of ERα by tamoxifen, a selective ER modulator and the standard first-line endocrine therapy for the past 30 years, effectively prevents the growth and metastasis of luminal breast cancer (57). Dwane *et al.* reported that USP11, among 108 deubiquitinases, functions as the most significant regulator of ERα transcriptional activity (72). The authors showed that the expression of USP11 and ERα is positively correlated in human BC lines, and knockdown of USP11 inhibits the expression of ERα target genes in ERα-positive human BCs; however, they did not determine whether USP11 regulates the expression of ERα *per se* (72). In this research, we provide the following genetic and molecular evidence to demonstrate the regulation of ERα expression by USP11: 1) both mRNA and protein levels of ERα were reduced in Usp11-null mammary epithelial cells (MEC); 2) knockdown of USP11 in human and mouse luminal-type mammary tumor cells reduced the expression of ERα; 3) overexpression of WT USP11, but not the enzyme-inactive C318A mutant, promoted the expression of ERα in human and mouse mammary tumor cells. Importantly, we discovered that USP11 regulates ERα through GATA3, providing a mechanism by which USP11 stimulates ERα expression. Although we did not determine how GATA3 regulates ERα, it has been shown that GATA3 promotes transcription of Esr1 (73). In addition, we not only confirmed the positive correlation between USP11 and ERα expression in human breast cancer samples, but also uncovered that ectopic USP11 sensitizes luminal-type BCs to tamoxifen treatment. Thus, the USP11-GATA3 signaling axis, through maintaining ERα expression, maintains and strengthens the luminal features of breast cancer, highlighting the potential diagnostic and therapeutic roles of this pathway in breast cancer therapy.

Our findings reveal the critical role of the USP11-GATA3 axis in maintaining luminal cell fate and restraining luminal cell proliferation in luminal breast cancer. This discovery carries several potential clinical implications. First, the positive correlation between USP11 expression and key luminal markers (GATA3, ERα) suggests that assessing USP11 levels could serve as a novel prognostic biomarker. Specifically, low USP11 expression might identify luminal breast cancer patients at higher risk of losing luminal features or developing resistance to endocrine therapy. Second, our observation that USP11 overexpression sensitizes luminal BCs to tamoxifen highlights USP11 as a potential predictive biomarker for endocrine therapy response. Patients with high USP11 expression may be more likely to benefit from tamoxifen. Third, due to its context-dependent role in luminal and basal-like tumorigenesis, non-selective agonism of USP11 should be avoided in treatment. We suggest that restoring or maintaining USP11 activity specifically in luminal tumors could help preserve luminal identity, prevent lineage switching, and overcome endocrine resistance. Future studies are warranted to develop small molecules that selectively enhance USP11's deubiquitinase activity or stabilize its interaction with GATA3, as well as to evaluate whether combining USP11 enhancement with tamoxifen improves clinical outcomes in patients with refractory or relapsed luminal breast cancer.

## Experimental procedures

### Mice, histopathology and immunostaining

The generation of Usp11 knockout mice on a C57BL/6J background was previously described (29). Female NCG mice were purchased from GemPharmatech. Mice were maintained in specific pathogen-free environment with a 12/12 light/dark cycle and allowed free access to water and food. All animal experiments were approved by the Institutional Animal Care and Use Committee at Shenzhen University. The investigators were not blinded to genotype allocation during experiments and outcome assessment. No randomization method was used as mice were segregated into groups based on genotype. Histopathology and immunostaining were performed as previously described (6, 74). Primary antibodies used for IHC staining were against GATA3 (R, #5852S, CST), USP11 (M, sc-365528), Ki-67 (R, #9129S, CST), ERα (R, GB111843, Servebio), and p27 (M, sc-1641). After primary antibody incubation, mammary gland or tumor sections were incubated with the MaxVision HRP-Polymer anti-Mouse/Rabbit IHC Kit (Maixin Biotech) and visualized using the DAB kit (Maixin Biotech) according to the manufacturer’s instructions. Cell nuclei were visualized by hematoxylin. The relative expression levels of GATA3, USP11, ERα, and p27 in human breast tumor cells were quantified by IHC-H score, as described previously (75). Primary antibodies used for immunofluorescent staining were against GATA3, p27, ERα, E-cad, and USP11. After primary antibody incubation, T47D or mouse mammary tumor cells were incubated with Alexa Fluor 488-conjugated anti-rabbit IgG (H + L) (#4412, CST) and Alexa Fluor 594-conjugated anti-mouse IgG (H + L) (#8890, CST) secondary antibodies. Cell nuclei were visualized by DAPI.

### Cell culture

Primary MECs prepared from 5-week-old *Usp11*^*+/+*^ and *Usp11*^*−/−*^ female mouse mammary glands were maintained in MM + medium as previously described (7). The human BC lines MCF-7, T47D, MDA-MB231, HCC1937, BT549, and BT20, as well as the mouse mammary tumor cell line PyMT-2095, and cells from *p18*^*+*^*^/^*^*−*^*;Brca1*^*+/−*^ (*Brca1*^*+/−*^ 1276 and *Brca1*^*+/−*^ 1291) and *p18*^*+*^*^/^*^*−*^*;Gata3*^*+/−*^ (*Gata3*^*+/−*^) mice, and 4T1 cells were maintained in DMEM/F12 medium with 10% fetal bovine serum as previously described (7, 29, 55, 74). For the luminal tumor cell lines T47D, MCF-7 and PyMT-2095, additional estrogen (1000 ng/ml, ST1101, Beyotime) was added to the medium. Human HEK293T cells purchased from ATCC were maintained in DMEM (#11965092, Gibco) medium with 10% fetal bovine serum.

### Gene knockdown and overexpression

For gene knockdown, 21-bp oligos targeting different sequences of the human *GATA3*, mouse *Gata3*, or *Cdkn1b* (p27Kip1) were constructed into the third-generation pLKO.1-puro vector (Addgene, Watertown, MA) as pLKO.1-puro-sh-GATA3#1 (sh-GATA3#1), pLKO.1-puro-sh-GATA3#2 (sh-GATA3#2), pLKO.1-puro-sh-Gata3 (sh-Gata3), and pLKO.1-puro-sh-p27 (sh-p27), separately. These shRNA sequences were obtained from the Hairpins RNAi Design Tools (https://www.broadinstitute.org/) and are listed as follows: human GATA3#1: 5′-GGAGAAATAGTGTGGAAATTA-3′; human GATA3#2: 5′-GACCGAACTGTTGTATAAATT-3′; mouse Gata3: 5′-TAACTGCAAACAAACCATTAA-3′; mouse p27: 5′-CCGCCATATTGGGCAACTAAA-3′. Gene knockdown oligo sequences for human *USP11*, mouse *Usp11*, and control were described previously (29), and were constructed into the pLKO.1-puro vector as pLKO.1-puro-sh-USP11#1 (sh-USP11#1), pLKO.1-puro-sh-USP11#2 (sh-USP11#2), pLKO.1-puro-sh-Usp11#1 (sh-Usp11#1), pLKO.1-puro-sh-Usp11#2 (sh-Usp11#2), and pLKO.1-puro-sh-Control (sh-Ctrl). cDNAs encoding USP11, USP11-C318A mutant, and GATA3 were cloned into the pLVX-IRES-Puro-3xFlag (Addgene) vector as pLVX-IRES-Puro-Flag-USP11 (Flag-USP11), pLVX-IRES-Puro-Flag-USP11-C318A (Flag-USP11-C318A), and pLVX-IRES-Puro-Flag-GATA3 (Flag-GATA3), respectively. The pLKO.1-puro or pLVX-IRES-Puro-3xFlag vector was used as the backbone plasmid and was co-transfected with the packaging plasmid psPAX2 (Addgene) and the envelope plasmid pMD2.G (Addgene), in a ratio of 9:6:3, into HEK293T cells to package lentivirus. After 2 days of lentivirus infection, mammary tumor cells were selected with 1000 ng/ml puromycin or 2000 ng/ml hygromycin for 5 days and then maintained in culture medium with 100 ng/ml puromycin or 200 ng/ml hygromycin for further use.

### Cell proliferation, scratch wound healing, and trans-well assays

For the cell proliferation assay, mouse primary MECls, PyMT-2095 tumor cells, and T47D tumor cells were seeded into 6-well plates at a density of 5000 cells/well. Two weeks later, the cells were harvested for counting and statistical analysis. For the cell scratch wound healing assay, T47D, MDA-MB231, or 4T1 cells were seeded into 6-well plates. When cells reached 100% confluence, scratches were made using a plastic disposable pipette tip (200 μl). Afterward, photos were taken at 0, 24, 48, and 72 h, and wound closures were quantified. For the cell trans-well assay, MECs and PyMT-2095 cells were seeded into the upper chamber at 10,000 cells per assay. The cell culture medium in the upper chamber was serum-free and in the lower chamber contained 10% FBS. After 24 h, the migrated cells on the bottom side of the membrane were fixed and stained with crystal violet, and then quantified.

### Transplantation model of mammary tumors and experimental metastasis model

For mammary transplantation, 5 × 10^5^ stable PyMT-2095 cells in Matrigel (356234, Corning) containing 2000 ng/ml estrogen were injected into the mammary fat pads of 5-week-old immunodeficient NCG mice. Twenty days post-transplantation, the generated tumors were dissected and analyzed. For the generation of an experimental metastasis model, 1 × 10^6^ stable PyMT-2095 cells were introduced into NCG mice through tail vein injection. Twenty-two days post-injection, mice were sacrificed and lung tissues were obtained for H&E staining. Metastatic lung nodules were quantified following our established protocol (76). In brief, all five lobes of fixed lung tissues were sagittally sliced at 200-μm intervals, with a minimum of three sections per lobe prepared and stained with H&E. The metastatic nodules in each lobe were verified *via* H&E staining, counted microscopically, and averaged, followed by calculation of the total nodule count across all lobes.

### Western blotting

Proteins for western blotting from cells, mammary glands, and tumors were prepared using SDS Lysis Buffer (P0013G, Beyotime) according to the manufacturer’s instructions. Primary antibodies used for western blotting were against USP11, GATA3, p27, ERα, E-cad, Vimentin (R, #5741S, CST), GST (R, AF2299, Beyotime), Ubiquitin (R, AF1705, Beyotime), p21 (R, sc-397), GAPDH (R, AF0006, Beyotime, China), HSP90 (R, AH732, Beyotime), and β-Actin (R, AF2811, Beyotime).

### Co-immunoprecipitation

For each immunoprecipitation, 2 mg of T47D cell lysates prepared using Cell Lysis Buffer for Western and IP (P0013, Beyotime) was incubated with 2 μg of anti-USP11 antibody (M), anti-GATA3 (R) antibody, anti-p27 (M), anti-ERα (R), mouse IgG (A7028, Beyotime), or rabbit IgG (A7016, Beyotime) at 4 °C overnight. The antibody and its conjugated proteins were precipitated using Protein A + G Agarose (P2055, Beyotime). USP11, GATA3, p27, and ERα protein levels in the immunoprecipitates and input samples were analyzed by immunoblotting.

### GST pull-down

The GST and GST-tagged N-terminus of USP11 (amino acids 1-300, termed GST-USP11-N) and C-terminus of USP11 (amino acids 301-963, termed GST-USP11-C) fusion proteins were prepared in BL21(DE3) *Escherichia coli* (D0337, Beyotime) as described previously (29). For the GST Pull-down assay, the same amount of GST, GST-USP11-N, or GST-USP11-C protein was incubated with T47D cell lysates containing endogenous GATA3 protein for 1 h. BeyoGold GST-tag Purification Resin (P2250, Beyotime) beads were then added to precipitate GST and GST fusion proteins together with their binding proteins. The precipitates were immunoblotted by using anti-GST and anti-GATA3 antibodies respectively.

### qRT-PCR

Total RNA was extracted from mammary glands, tumors, and cells using TaKaRa RNAiso Reagent (TaKaRa Biotechnology Co) and reverse-transcribed into cDNA with the BeyoRT III First Strand cDNA Synthesis Kit (D7178S, Beyotime) according to the manufacturer’s instructions. The expression levels of *USP11*, *GATA3*, *CDKN1B*, *Gata3*, *Cdkn1a*, *Cdkn1b*, and *Esr1* were determined using BeyoFast SYBR Green qPCR Mix (D7260, Beyotime) according to the manufacturer’s instruction. Primers for qRT-PCR are listed in Table S1.

### Ubiquitin assay

T47D cells were infected with Flag, Flag-USP11, Flag-USP11-C318A, sh-Ctrl, sh-USP11#1, or sh-USP11#2 lentivirus separately for 72 h. Cell lysates were prepared and incubated with GATA3 antibody for immunoprecipitation. The levels of ubiquitinated GATA3 in the precipitates and the levels of GATA3 and USP11 in the cell lysates were immunoblotted.

### Chromatin-immunoprecipitation assay

Chromatin-immunoprecipitation assays were performed as described previously (59). Briefly, T47D cells at 80% density were treated with formaldehyde and sonicated, and then chromatin associated with GATA3 was immunoprecipitated using anti-GATA3 antibody or control rabbit IgG. The abundances of four predicted GATA3 binding sites, P1 (+34 bp to +40 bp), P2 (−1681 bp to −1675 bp), P3 (−2094 bp to −2088 bp) and P4 (−4774 bp to −4768 bp), within the *CDKN1B* gene region were examined using specific primers by qPCR. Primers used for qPCR are listed in Table S2.

### Dual-luciferase reporter assay

The human *CDKN1B* promoter region from −2715 bp to −1587 bp covering P2 and P3 was inserted into the pGL3-basic vector (Promega) to generate pGL3-*CDKN1B*. For the promoter-luciferase reporter assay, HEK293T cells were transfected with pGL3-*CDKN1B*, Renilla, PLVX-Flag (Empty), PLVX-Flag-USP11, PLVX-Flag-USP11-C318A, PLVX-Flag-GATA3, PLKO-Sh-Ctrl, or PLKO-Sh-GATA3 plasmids for 48 h. Cell lysates were collected and subjected to a luciferase assay using the Dual-Luciferase Reporter Assay System (Promega) following the manufacturer’s instructions.

### Human tumor samples and meta-analysis of gene expression data sets

De-identified paraffin-embedded human breast tumor sections were provided by the Gansu Dian Medical Laboratory. The expression of USP11, GATA3, ERα, and p27 in breast tumor cells was assessed by IHC staining and quantified on four serial sections of each human tumor respectively. The correlations between USP11 & GATA3, USP11 & p27, USP11 & ERα, GATA3 & p27, and GATA3 & ERα were statistically analyzed based on the IHC H-score. The correlation between USP11 expression levels and overall survival of patients was analyzed in a cohort of breast cancer patients treated with tamoxifen, using dataset GSE58644 from the public GEO Datasets (58). Patients were stratified into high and low USP11 expression groups based on the median expression level. Overall survival was measured from the initiation of tamoxifen treatment, with follow-up data available for up to 150 months. Kaplan–Meier survival curves were generated, and the log-rank test was used to compare survival distributions between the two groups.

### Statistical analysis

Two-tailed Student's *t* test was used for pairwise comparisons in quantitative results. Data are presented as the mean ± SEM from three or more repeated independent experiments. *p* <0.05 was considered statistically significant.

### Ethics declaration

The Institutional Animal Care and Use Committee at Shenzhen University approved all animal procedures.

## Consent for publication

Not applicable.

## Data availability

All the presented data are available within the article or supplementary files.

## Supporting information

This article contains supporting information.

## Conflict of interest

The authors declare that they have no conflicts of interest with the contents of this article.
