# Supplementary material for: USP11 deubiquitinates GATA3 to suppress the growth and metastasis of luminal-type breast cancers
Source: J Biol Chem. 2026 Jul 29;302(9):113371. doi: 10.1016/j.jbc.2026.113371 (PMC13524610; doi:10.1016/j.jbc.2026.113371)
Supplement: Supplementary file 1 [file mmc1.pdf]

Supplementary table 1

Primers for qRT-PCR

Species	Gene	Primers
Human	*USP11*	Forword: 5’-TATAAGCAGTGGGAGGCATACG-3’<br>Reverse: 5’-ATGACCTTGCGTTCAATGGGT-3’
Human	*GATA3*	Forword: 5’-CACAACCACACTCTGGAGGAG-3’<br>Reverse: 5’-GTCCTCCAGTGAGTCATGCAC-3’
Human	*CDKN1B*	Forword: 5’-CGGCTAACTCTGAGGACACG-3’<br>Reverse: 5’-AGAATCGTCGGTTGCAGGTC-3’
Mouse	*Gata3*	Forword: 5’-GCTGACGGAAGAGGTGGACGTACT-3’<br>Reverse: 5’-TGGTGGGTCGGAGGATACCT-3’
Mouse	*Cdkn1a*	Forword: 5’-TAAGGACGTCCCACTTTGCC-3’<br>Reverse: 5’-CAGGATTGGACATGGTGCCT-3’
Mouse	*Cdkn1b*	Forword: 5’-ACAATCCGGCTGGGTTAGC-3’<br>Reverse: 5’-TTTACGTCTGGCGTCGAAGG-3’
Mouse	*Esr1*	Forword: 5’-GCCCTACTACCTGGAGAACGA-3’<br>Reverse: 5’-CAGTCTCTCTCGGCCATTCTG-3’

Supplementary table 2

Primers for ChIP assay

Fragments	Predicated binding site	Primers
GATA3-P1	34/40bp	Forword: 5’-ACCTTAAGGCCGCGCTCGCC-3’<br>Reverse: 5’-AAAACACCCCGAAAAGACGA-3’
GATA3-P2	-1681/-1675b	Forword: 5’-ACATCAAGGAAACATCTGCAG-3’<br>Reverse: 5’-GATGCAAGTCTCCTAACACT-3’
GATA3-P3	-2094/-2088bp	Forword: 5’-ACATACATGTGGTAGTTTGG-3’<br>Reverse: 5’-TGTCAATTTATTGGACTGCA-3’
GATA3-P4	-4774/-4768bp	Forword: 5’-ATGCCATCCATGGCCAACCA-3’<br>Reverse: 5’-GATAGAGCAAGACATTGTCT-3’

Supplementary figure 1

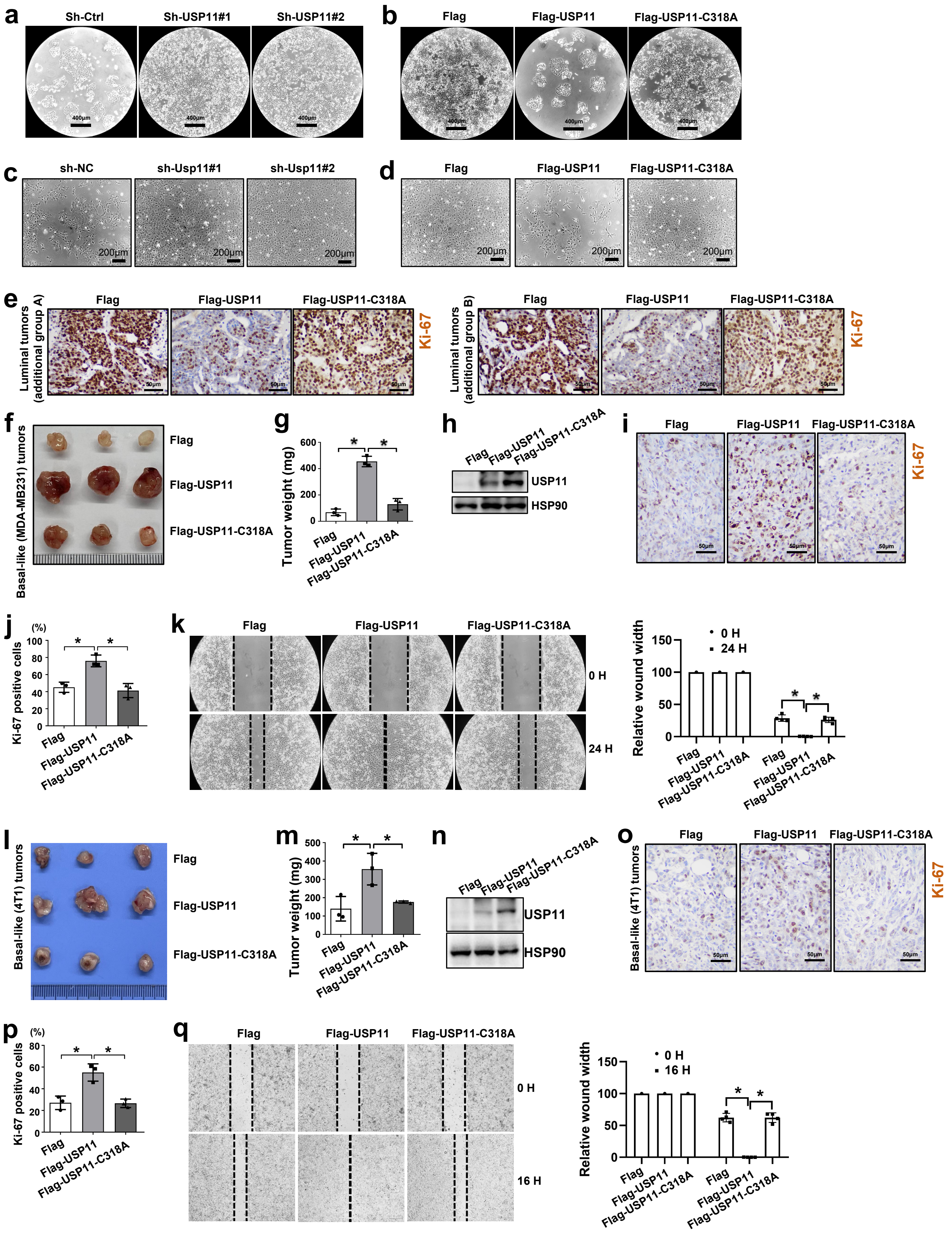

Figure S1. USP11 suppresses luminal, but promotes basal-like, breast cancer cell proliferation and migration, as well as tumor growth

(**a, b**) Phase contrast imaging of T47D cells infected with Sh-Ctrl, Sh-USP11#1, or Sh-USP11#2 (**a**) or Flag, Flag-USP11, or Flag-USP11-C318 (**b**) for 48 hours; (**c, d**) Phase contrast imaging of PyMT-2095 cells infected with Sh-Ctrl, Sh-Usp11#1, or Sh-Usp11#2 (**c**) or Flag, Flag-USP11, or Flag-USP11-C318 (**d**) for 48 hours. In **a-d**, 10,000 cells per well were seeded. (**e**) IHC staining of Ki-67 in additional tumors described in (**Fig.1t**) (**f-j**) Gross appearance (**f**) and the weight (**g**) of the tumors generated by MDA-MB231 cells stably overexpressing Flag, Flag-USP11, or Flag-USP11-C318. n=3; *P<0.05. Representative western blot (**h**) and IHC (**i**) analysis of the tumors described in (**f**). (**j**) Quantification of Ki-67-positive tumor cells in (**i**). n=3; *P<0.05. (**k**) (left) Representative photos from scratch healing assay of MDA-MB231 cells infected with Flag, Flag-USP11, or Flag-USP11-C318; (right) the gap closure in percent was determined at the 0- and 24-hour, n=4, *P<0.05. (**l-p**) Gross appearance (**l**) and the weight (**m**) of the tumors generated by 4T1 cells stably overexpressing Flag, Flag-USP11, or Flag-USP11-C318. n=3; *P<0.05. Representative western blot (**n**) and IHC (**o**) analysis of the tumors described in (**l**). (p) Quantification of Ki-67-positive tumor cells in (**o**). n=3; *P<0.05. (**q**) (left) Representative photos from scratch healing assay of 4T1 cells infected with Flag, Flag-USP11, or Flag-USP11-C318; (right) the gap closure in percent was determined at the 0- and 16-hour. n=4, *P<0.05.

Supplementary figure 2

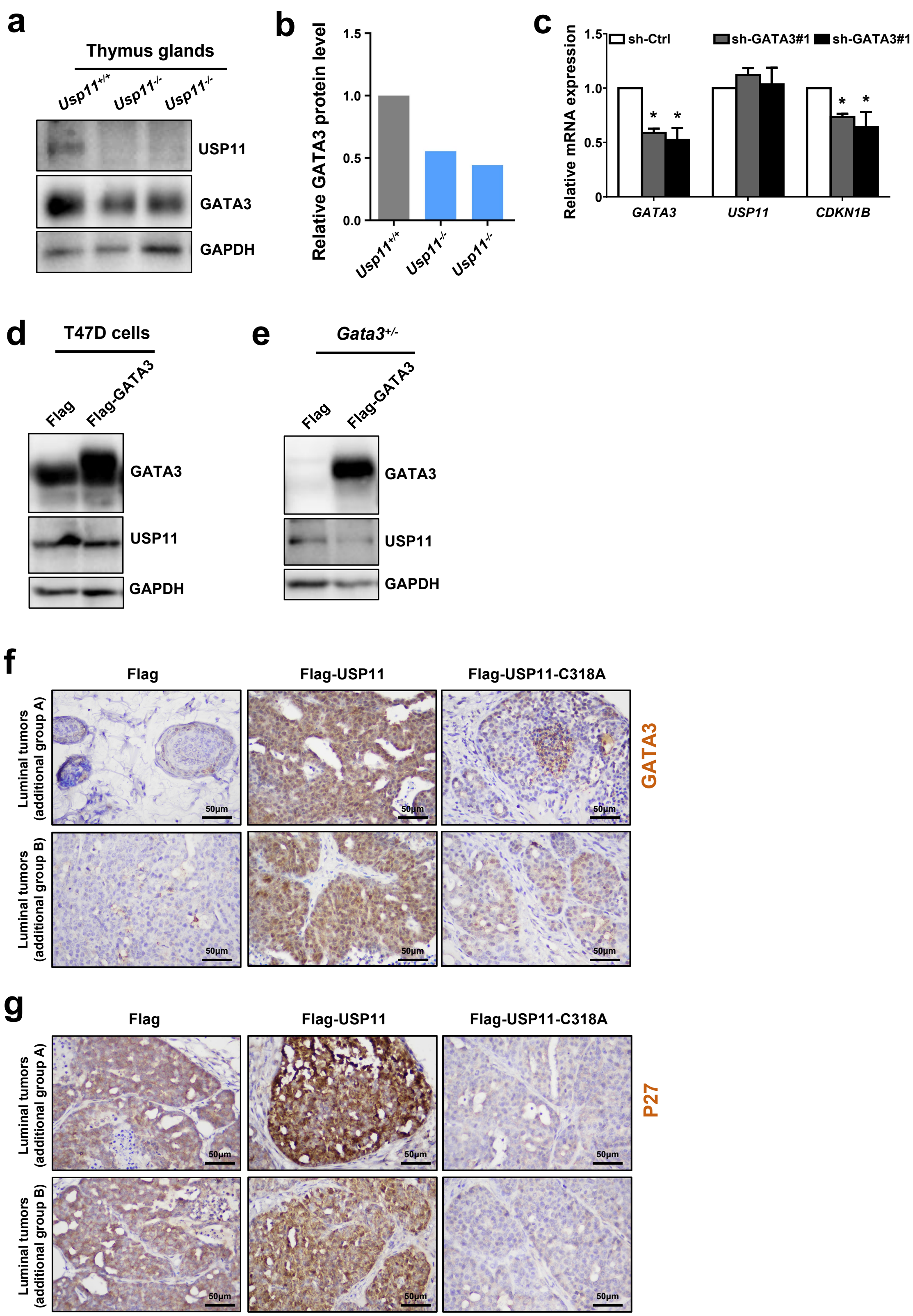

Figure S2. USP11 maintains the expressions of GATA3 and p27 in thymocyte and mammary cells, whereas GATA3 has minimal impact in regulating USP11

(**a, b**) The expression of USP11 and GATA3 in thymus from 5-week-old *Usp11*$^{+/+}$ and *Usp11*$^{-/-}$ mouse were immunoblotted (**a**) and quantified (**b**), with GAPDH serving as an internal control. (**c**) T47D cells were infected with Sh-Ctrl, Sh-GATA3#1, or Sh-GATA3#2 lentivirus for 48 hours. The mRNA expressions of USP11, GATA3, and CDKN1B were determined by qRT-PCR. n=4; *P<0.05. (**d, e**) T47D (d) and *Gata3*$^{+/-}$ (**e**) breast tumor cells were infected with Flag or Flag-GATA3 lentivirus for 48 hours. The expression of GATA3 and USP11 were immunoblotted, with GAPDH serving as internal control. (**f, g**) Representative IHC analysis of GATA3 (**f**) and p27 (**g**) in additional tumors described in (**Fig.1s**).

Supplementary figure 3

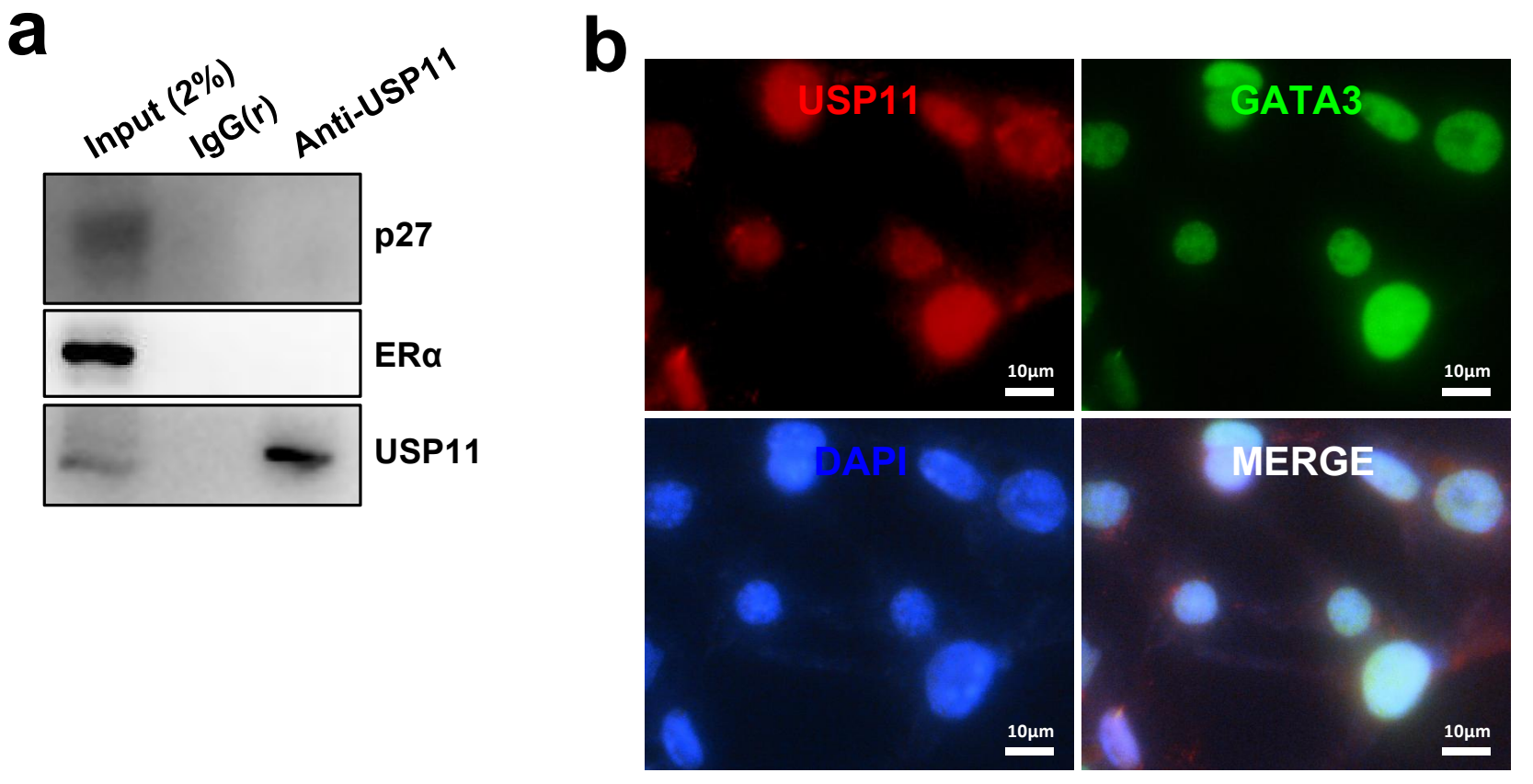

Figure S3. USP11 binds to GATA3, but not p27

(**a**) T47D cell lysates were immunoprecipitated with USP11 antibody or control IgG, and the expressions of p27, ERα, and USP11 in input and immunoprecipitated samples were detected by western blotting. (**b**) Representative immunofluorescent staining of USP11 (red) and GATA3 (green) in PyMT-2095 cells. DAPI was used to visualize the cell nucleus.

Supplementary figure 4

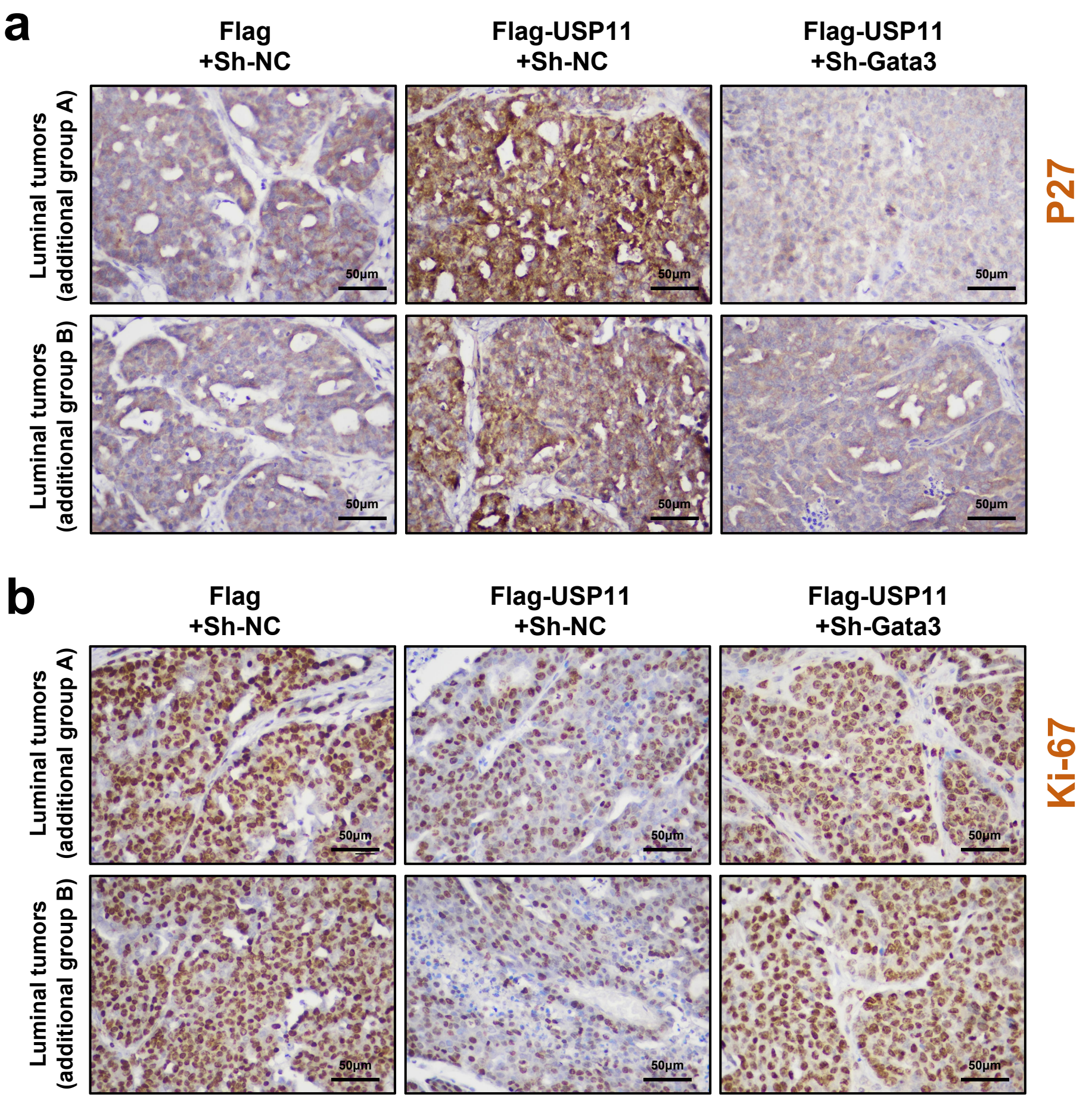

Figure S4. IHC analysis of p27 and Ki-67 in additional tumors generated by luminal tumor cells expressing USP11 with or without GATA3 knockdown (KD)

Representative IHC analysis was performed to detect the expression of p27 (**a**) and Ki - 67 (**b**) in tumors. These tumors were generated by PyMT-2095 cells infected with different combinations of lentivirus: either Flag or Flag-USP11, along with either Sh-NC or Sh-Gata3.

Supplementary figure 5

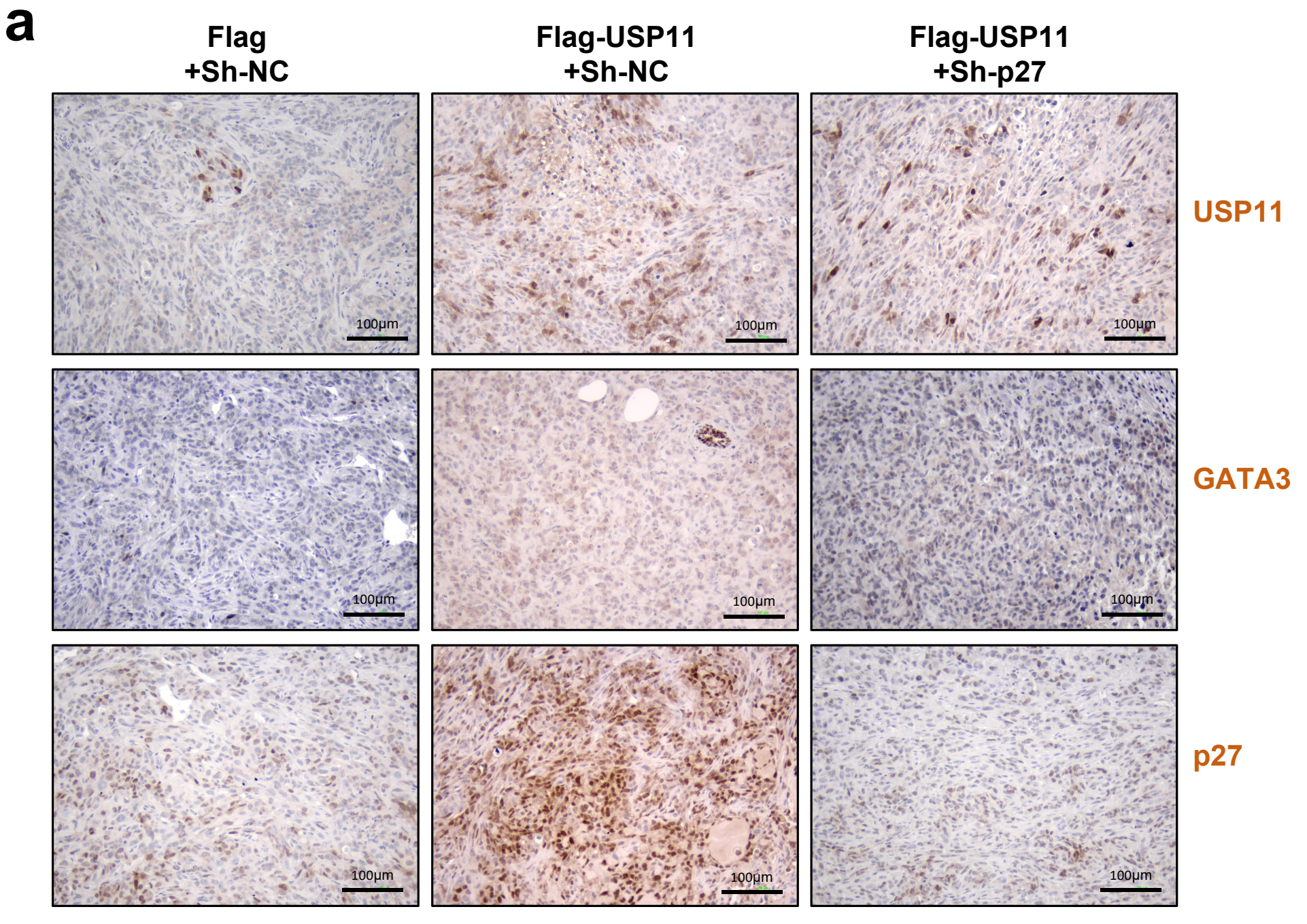

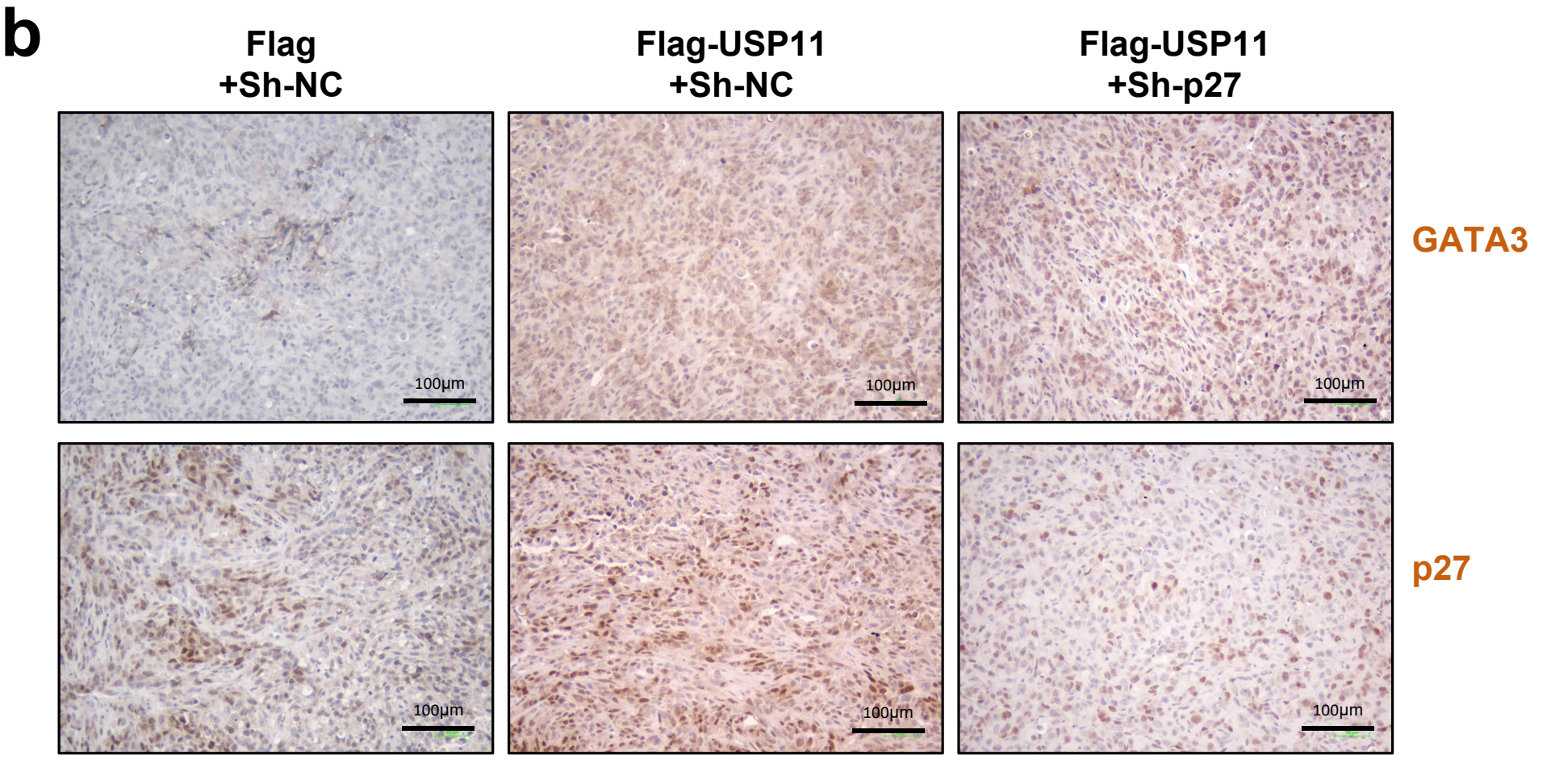

Figure S5 IHC analysis of additional tumors generated by luminal tumor cells expressing USP11 or GATA3 with or without p27 KD

(**a, b**) Representative IHC analysis was performed to detect the expression of USP11, GATA3, or p27 in tumors. These tumors were generated by PyMT-2095 cells infected with different combinations of lentivirus: either Flag, Flag-USP11, or Flag-GATA3, along with either Sh-NC or Sh-p27.
